# Cavity Quantum Electrodynamic Nuclear–Electronic Orbital Complete Active Space Configuration Interaction

**DOI:** 10.1021/acs.jctc.6c01384

**Published:** 2026-10-24

**Authors:** Scott M. Garner, Sharon Hammes-Schiffer

**Affiliations:** Department of Chemistry, 6740 Princeton University, Princeton New Jersey 08544, United States

## Abstract

Polaritons offer the possibility of modifying chemical properties through the strong coupling of molecules to photons confined in optical cavities. From a first-principles perspective, understanding how these strong light–matter interactions cause chemical changes requires quantum mechanical descriptions of electrons, nuclei, and photons. Herein, we develop the ab initio cavity quantum electrodynamic nuclear–electronic orbital complete active space configuration interaction (QED-NEO-CASCI) method, in which electrons, specified nuclei, and cavity photons are all treated quantum mechanically. Using this method, we are able to describe hydrogen tunneling under vibrational strong coupling and geometry changes in a hydrogen-bonded complex coupled to a cavity. Under vibronic strong coupling, we observe features in the polaritonic absorption spectrum that correspond to Raman active transitions outside a cavity. We also develop the real-time variant, denoted time-dependent configuration interaction (QED-NEO-TDCI). This method allows us to simulate nonadiabatic hydrogen tunneling dynamics and observe the real-time quantum mechanical transfer of energy between the molecule and cavity photons. These methods provide the ab initio foundation for studying correlated electronic–nuclear–photonic chemical physics.

## Graphical abstract

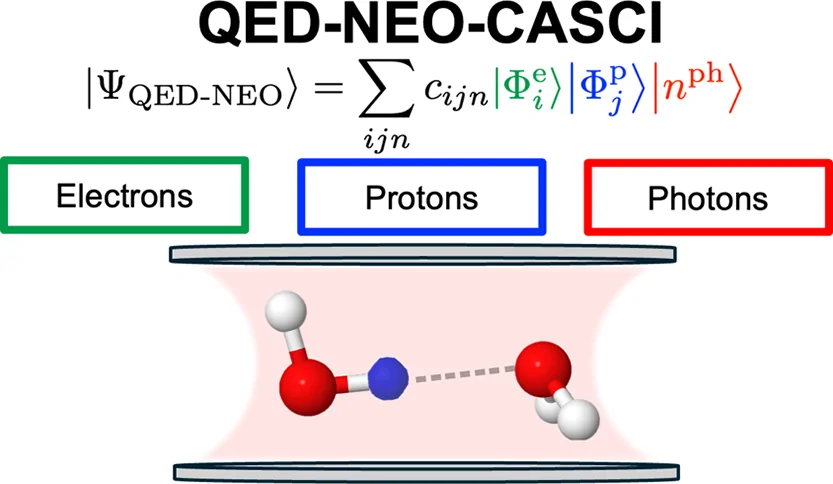

## 1 Introduction

The coupling of molecules to optical cavities creates hybrid light–matter states called polaritons.^1,2^ Coupling can be achieved by tuning the cavity optical modes to be resonant with molecular electronic or vibrational transitions. These two cases correspond to electronic strong coupling (ESC) and vibrational strong coupling (VSC), respectively. Polaritons have gained significant interest due to a large number of possible applications,^1^ including the proposal for using polaritons to control chemical reactivity.^3–7^

Modeling the interaction between molecules and cavity photons has presented an interesting challenge for the theoretical physics and chemistry communities. Although a significant number of theoretical approaches have been investigated and reviewed,^8–11^ our focus here is on ab initio quantum electrodynamic (QED) methodology. QED methods are a subset of multicomponent quantum chemistry, where more than one type of particle is treated quantum mechanically, in this case both electrons and photons.^10,12–14^ The quantum mechanical treatment of electrons and cavity photons enables first-principles studies of how coupling molecules to cavities alters chemical reactivity, including proton transfer,^15,16^ the Diels–Alder reaction,^17^ and cycloaddition reactions.^18^ Numerous tools from molecular quantum chemistry have been adapted to implement QED analogs, including density functional theory (QEDFT)^19–21^ and correlated wave function theories such as configuration interaction singles (QED-CIS)^22^ and related spin-flip CI (QED-SF-CIS),^23^ complete active space self-consistent field (QED-CASSCF),^24,25^ coupled cluster theory (QED-CC),^26–29^ density matrix renormalization group (QED-DMRG),^30^ time-dependent configuration interaction (QED-TDCI),^31,32^ and various flavors of quantum Monte Carlo (QED-QMC).^33,34^

Of particular relevance to this work is QED complete active space configuration interaction (QED-CASCI).^26,29,35^ A QED-CASCI wave function for a single cavity mode is expressed as

1 $$\left|\Psi_{\mathrm{QED}‐\mathrm{CASCI}}\right\rangle =\underset{in}{\sum }c_{in}\left|\Phi_{i}^{e}\right\rangle \left|n^{\mathrm{ph}}\right\rangle$$

and is formed by taking the direct product of electronic Slater determinants, $\left|\Phi_{i}^{e}\right\rangle$ , and photon number Fock states, $\left|n^{\mathrm{ph}}\right\rangle$ , where *n* is the number of photons occupying the cavity mode. The wave function expansion coefficients, *c*_*in*_, are obtained by diagonalizing a joint light–matter Hamiltonian. QED-CASCI calculations produce wave functions that are easily interpreted and can be used to capture strong correlations between electrons and photons under ESC.^35^

Another multicomponent quantum chemistry approach is the nuclear–electronic orbital (NEO) method,^36–38^ where specified nuclei, typically protons, are treated quantum mechanically on the same level as the electrons using wave function or density functional theories. The NEO framework has similarly been implemented using a wide variety of quantum chemistry methods, including density functional theory (NEO-DFT)^39,40^ and correlated wave function methods, such as complete active space self-consistent field (NEO-CASSCF),^36,41^ multireference configuration interaction (NEO-MRCI),^42^ and coupled cluster theory (NEO-CC).^43–47^ Similar to QED-CASCI, a NEO-CASCI wave function is expressed as

2 $$\left|\Psi_{\mathrm{NEO}‐\mathrm{CASCI}}\right\rangle =\underset{ij}{\sum }c_{ij}\left|\Phi_{i}^{e}\right\rangle \left|\Phi_{j}^{p}\right\rangle$$

where $\left|\Phi_{i}^{e}\right\rangle$ is again an electronic Slater determinant and, analogously, $\left|\Phi_{j}^{p}\right\rangle$ is a nuclear Slater determinant. The wave function expansion coefficients, *c*_*ij*_, are obtained by diagonalizing the NEO Hamiltonian, which in addition to the standard electronic Hamiltonian contains the kinetic energy of the quantized nuclei and all Coulomb interactions among quantum and classical particles.

Several real-time NEO (RT-NEO) methods,^48–50^ where the time-dependent Scrödinger equation is numerically propagated, have also been developed to simulate dynamics. Combining RT-NEO methods with a cavity mode has provided an avenue to evenhandedly study both ESC and VSC. Using a classical description of the cavity mode with RT-NEO time-dependent DFT (RT-NEO-TDDFT)^51–53^ and NEO time-dependent configuration interaction (NEO-TDCI)^54^ provides semiclassical descriptions of spectroscopy and dynamics for ESC and VSC. Moreover, the semiclassical NEO-TDCI approach captures vibronic strong coupling, where the cavity mode couples to a progression of joint electronic−nuclear excitations rather than a bare electronic transition.^54^ These semiclassical methods do not treat the cavity mode quantum mechanically. Additionally, only time-dependent properties, such as the time-dependent molecular dipole moment and classical cavity displacement, are available from these methods, while the underlying time-independent polaritonic wave functions are not obtained directly. A full quantum treatment of the cavity mode in RT-NEO-TDDFT has been investigated,^52,55^ although this tool is somewhat limited in scope due to the inherent limitations of RT-NEO-TDDFT and still lacks access to time-independent polaritonic wave functions.

To address these limitations, in this work, we combine QED and NEO methodologies to perform ab initio QED-NEO calculations, where electrons, specified nuclei, and photons are all simultaneously treated quantum mechanically. We first provide the working equations for QED-NEO Hartree–Fock (QED-NEO-HF), where the interactions among electrons, quantum protons, and photons are all treated at the mean-field level. We then present our primary methodological contribution, which is QED-NEO-CASCI. Within QED-NEO-CASCI, our wave function will be expressed as

3 $$\left|\Psi \right\rangle =\underset{ijn}{\sum }c_{ijn}\left|\Phi_{i}^{e}\right\rangle \left|\Phi_{j}^{p}\right\rangle \left|n^{\mathrm{ph}}\right\rangle$$

where $\left|\Phi_{i}^{e}\right\rangle$ and $\left|\Phi_{j}^{p}\right\rangle$ are individual electronic and protonic Slater determinants, respectively, and $\left|n^{\mathrm{ph}}\right\rangle$ is a photon number Fock state corresponding to *n* photons in the cavity mode. The wave function coefficients are obtained by diagonalizing a joint light–matter Hamiltonian that treats the molecular degrees of freedom with the NEO Hamiltonian. In this manner, within the active spaces for electrons, quantized nuclei, and photons, all many-body interactions are treated exactly quantum mechanically. Using our QED-NEO-CASCI implementation, we study two molecular systems under VSC, the HeHHe^+^ molecular cation and a water dimer, as well as HeHHe^+^ under vibronic strong coupling. We analyze the strong correlations between the quantized nuclei and photons by directly accessing the time-independent ab initio QED-NEO-CASCI wave functions. Additionally, we also present the real-time variant, QED-NEO-TDCI, which enables us to simulate proton tunneling dynamics in HeHHe^+^ and observe the quantum mechanical exchange of energy among the electronic, protonic, and photonic degrees of freedom in real time.

The remainder of this paper is organized as follows. Section 2 presents the theoretical framework for the ab initio QED-NEO methodology, particularly focusing on the NEO-specific alterations of QED methods. Section 3 provides the computational details used in our QED-NEO-CASCI and QED-NEO-TDCI calculations. Section 4.1 builds the framework for analyzing our time-independent wave functions through VSC calculations of the HeHHe^+^ model system. Section 4.2 presents simulations of real-time hydrogen tunneling dynamics of HeHHe^+^ under VSC, and Section 4.3 shows vibronic strong coupling, where the cavity is coupled to a progression of vibrational states associated with an electronic excited state in HeHHe^+^. Section 4.4 presents results displaying geometric effects under VSC for a quantum proton involved in the hydrogen-bonding interaction within a water dimer. Finally, Section 5 provides a summary of our results and concluding remarks.

## 2 Theory

### 2.1 Hamiltonian

We begin with the Pauli–Fierz (PF) Hamiltonian within the length gauge and dipole approximations:^13,14,17,18,21,56^

4 $${Ĥ}_{\mathrm{PF}}={Ĥ}_{\mathrm{mol}}+\underset{i}{\sum }\left[\omega_{i}b_{i}^{†}b_{i}-\sqrt{\frac{\omega_{i}}{2}}\lambda_{i}\cdot \mathrm{\mu ̂}\left(b_{i}+b_{i}^{†}\right)+\frac{1}{2}{\left(\lambda_{i}\cdot \mathrm{\mu ̂}\right)}^{2}\right]$$

where ω_*i*_ is the frequency, $b_{i}^{†}$ and *b*_*i*_ are the bosonic creation and annihilation operators, **λ**_*i*_ is the light–matter coupling vector for the *i*th cavity mode, and μ̂ is the molecular dipole operator. Equation 4 is written for a general multimode cavity. However, the current work is restricted to a single cavity mode with frequency ω, although the extension to multiple cavity modes is straightforward.^17,18^ In eq 4, the first term, ${Ĥ}_{\mathrm{mol}}$ , is the molecular Hamiltonian; the second term, *ωb* ^†^ *b*, is the energy of a bare harmonic cavity photon; the third term is the bilinear coupling, ${Ĥ}_{\mathrm{blc}}$ , between the cavity mode and molecular dipole operator; and the final term is the self-dipole term. In this work, all equations are given in atomic units, and we will work in Cartesian coordinates such that **λ** and μ̂ are vectors with *x*, *y*, and *z* components.

Our molecular Hamiltonian is the NEO Hamiltonian, which is expressed as

5 $${Ĥ}_{\mathrm{NEO}}={\mathrm{T̂}}_{e}+{\mathrm{V̂}}_{\mathrm{eN}}+{\mathrm{V̂}}_{\mathrm{ee}}+{\mathrm{T̂}}_{p}+{\mathrm{V̂}}_{\mathrm{pN}}+{\mathrm{V̂}}_{\mathrm{pp}}+{\mathrm{V̂}}_{\mathrm{ep}}$$

This Hamiltonian contains electronic (quantized nuclear) kinetic energies, ${\mathrm{T̂}}_{e\left(p\right)}$ ; electron (quantized nucleus)−classical nucleus Coulomb interactions, ${\mathrm{V̂}}_{\mathrm{eN}\left(\mathrm{pN}\right)}$ ; electron–electron (quantized nucleus−quantized nucleus) Coulomb interactions, ${\mathrm{V̂}}_{\mathrm{ee}\left(\mathrm{pp}\right)}$ ; and electron−quantized nucleus Coulomb interactions, ${\mathrm{V̂}}_{\mathrm{ep}}$ . We will use p as the label for quantized nuclei for this work because all of our quantum nuclei are protons, which typically exhibit the greatest nuclear quantum effects, although in principle other nuclei may also be treated quantum mechanically. The dipole operator for NEO calculations contains contributions from electrons, quantized nuclei, and classical nuclei:

6 $$\mathrm{\mu ̂}={\mathrm{\mu ̂}}_{e}+{\mathrm{\mu ̂}}_{p}+\mu_{N}$$

This NEO Hamiltonian invokes the Born–Oppenheimer approximation between the classical nuclei and the quantum nuclei and electrons, and the classical nuclei are fixed for the calculations presented herein. Thus, the classical nuclear contribution, **μ**_N_, is a constant vector.

### 2.2 QED-NEO-Hartree–Fock

Although not the focus of this work, we present a brief section outlining QED-NEO-HF theory, where the interactions between all quantum particles are treated as mean-field interactions. This theory provides the foundation for the correlated QED-NEO-CASCI theory that is the focus of this work. A QED-NEO-HF wave function is written as

7 $$\left|0\right\rangle =\left|0^{e}\right\rangle \left|0^{p}\right\rangle \left|0^{\mathrm{ph}}\right\rangle$$

where $\left|0^{e}\right\rangle$ and $\left|0^{p}\right\rangle$ are single electronic and nuclear Slater determinants composed of molecular orbitals expanded in Gaussian basis sets. The photon wave function, $\left|0^{\mathrm{ph}}\right\rangle$ , is a linear combination of cavity Fock states:^13,26^

8 $$\left|0^{\mathrm{ph}}\right\rangle =\underset{n}{\sum }c_{n}{\left(b^{†}\right)}^{n}\left|0\right\rangle$$

where |0⟩ is the photon vacuum. In QED-NEO-HF, the electronic molecular orbitals, quantum nuclear orbitals, and cavity wave function coefficients (*c*_*n*_ in eq 8) are all obtained by solving the time-independent Schrödinger equation.

The most straightforward approach for QED-NEO-HF calculations is to iteratively solve for the wave function associated with each type of quantized particle. Note that the molecular component of the total QED-NEO-HF wave function is simply a NEO-HF wave function, which is written as $\left|0^{\mathrm{NEO}}\right\rangle =\left|0^{e}\right\rangle \left|0^{p}\right\rangle$ . With this in mind, the electronic and nuclear wave functions can be jointly optimized, taking advantage of simultaneous optimizations available in many quantum chemistry codes with NEO-HF implementations.^57,58^ In the iterative approach, the NEO wave function is obtained by diagonalizing the PF Hamiltonian using the photon displacement expectation value for the current iteration. The molecule Hamiltonian is given as

9 $${Ĥ}_{\mathrm{PF}}^{\left\langle \mathrm{ph}\right\rangle }={Ĥ}_{\mathrm{NEO}}-\sqrt{\frac{\omega }{2}}\left(\lambda \cdot \mathrm{\mu ̂}\right)\left(\left\langle b\right\rangle +\left\langle b^{†}\right\rangle \right)+\frac{1}{2}{\left(\lambda \cdot \mathrm{\mu ̂}\right)}^{2}$$

where ⟨b⟩ and $\left\langle b^{†}\right\rangle$ are the expectation values of the cavity annihilation and creation operators. In eq 9, the NEO Hamiltonian term produces identical electronic and protonic Fock matrices as does NEO-HF theory.^36^ The bilinear coupling terms are then added to the electronic and protonic Fock matrices as an effective electric field in the direction of **λ** with strength $-\left|\lambda \right|\sqrt{\frac{\omega }{2}}\left(\left\langle b\right\rangle +\left\langle b^{†}\right\rangle \right)$ interacting with the molecular dipole. The self-dipole terms are also added to the respective Fock matrices and are computed as discussed in Section 2.3.

The photon wave function is then obtained by diagonalizing the Hamiltonian defined in terms of the molecular dipole expectation value:

10 $${Ĥ}_{\mathrm{PF}}^{\left\langle \mathrm{mol}\right\rangle }=\omega b^{†}b-\sqrt{\frac{\omega }{2}}\left(\lambda \cdot \left\langle \mathrm{\mu ̂}\right\rangle \right)\left(b+b^{†}\right)$$

where ⟨μ̂⟩ is the total molecular dipole operator expectation value. Equations 9 and 10 are solved self-consistently. We will refer to this procedure as photon-number (PN) QED-NEO-HF because the photon wave function is expressed in the basis of photon-number states.

An alternative approach for QED-NEO-HF is to use a coherent state (CS) transformation of the photon wave function:^26^

11 $$\left|0_{\mathrm{CS}}^{\mathrm{ph}}\right\rangle =\exp\left[\frac{-\lambda \cdot \left\langle \mathrm{\mu ̂}\right\rangle }{\sqrt{2\omega }}\left(b-b^{†}\right)\right]\left|0\right\rangle$$

where ⟨μ̂⟩ is again the total molecular dipole operator expectation value. Taking the expectation value of the PF Hamiltonian with respect to the coherent state photon wave function given in eq 11 and folding the exponential operator into the Hamiltonian gives the CS Hamiltonian expressed as^13,26^

12 ĤCS=⟨0CSph|ĤPF|0CSph⟩=ĤNEO+ωb†b−ω2[λ·(μ̂−⟨μ̂⟩)](b+b†)+12[λ·(μ̂−⟨μ̂⟩)]2

Note that the CS Hamiltonian is used to obtain only the NEO wave function, $\left|0^{\mathrm{NEO}}\right\rangle =\left|0^{e}\right\rangle \left|0^{p}\right\rangle$ . The CS formulation offers a few advantages over the PN formulation. First, the photon wave function is defined according to eq 11, avoiding the need to solve for the photon wave function (i.e., avoiding diagonalization of eq 10). Additionally, the bilinear coupling does not directly influence the electronic and protonic Fock matrices because the third term in eq 12 vanishes when operating on the NEO wave function. The only modifications to these Fock matrices are from the self-dipole term, again discussed in Section 2.3. Next, the subtraction of the dipole expectation value from the dipole operator alleviates origin dependence of the total energy for charged species.^13^ Finally, the dipole operator no longer needs to include contributions from the classical nuclei in the bilinear coupling or self-dipole terms, as $\mu_{N}=\left\langle \mu_{N}\right\rangle$ within the NEO Born–Oppenheimer approximation.

### 2.3 QED-NEO Self-Dipole Term

The self-dipole term, $\frac{1}{2}{\left(\lambda \cdot \mathrm{\mu ̂}\right)}^{2}$ in the PN-QED-NEO-HF formulation or $\frac{1}{2}{\left[\lambda \cdot \left(\mathrm{\mu ̂}-\left\langle \mathrm{\mu ̂}\right\rangle \right)\right]}^{2}$ in the CS-QED-NEO-HF formulation, requires special consideration as it differs from conventional ab initio QED methodology because the dipole operator includes contributions from both electrons and quantized nuclei. Starting with PN-QED-NEO-HF, expanding the self-dipole term with respect to the contributions from all types of particles to the dipole operator gives

13 $$\frac{1}{2}{\left(\lambda \cdot \mathrm{\mu ̂}\right)}^{2}=\frac{1}{2}{\left(\lambda \cdot {\mathrm{\mu ̂}}_{e}\right)}^{2}+\frac{1}{2}{\left(\lambda \cdot {\mathrm{\mu ̂}}_{p}\right)}^{2}+\left(\lambda \cdot {\mathrm{\mu ̂}}_{e}\right)\left(\lambda \cdot {\mathrm{\mu ̂}}_{p}\right)+\left(\lambda \cdot {\mathrm{\mu ̂}}_{e}\right)\left(\lambda \cdot \mu_{N}\right)+\left(\lambda \cdot {\mathrm{\mu ̂}}_{p}\right)\left(\lambda \cdot \mu_{N}\right)+\frac{1}{2}{\left(\lambda \cdot \mu_{N}\right)}^{2}$$

The first two terms of eq 13, corresponding to the self-dipole electronic and protonic terms, are expanded in second quantization^13^ as

14 $${\left(\lambda \cdot {\mathrm{\mu ̂}}_{e\left(p\right)}\right)}^{2}=\underset{\mu \nu \zeta \eta }{\sum }d_{\mu \nu }^{e\left(p\right)}d_{\zeta \eta }^{e\left(p\right)}{â}_{\mu }^{†}{â}_{\zeta }^{†}{â}_{\eta }{â}_{\nu }+Q\underset{\mu \nu }{\sum }q_{\mu \nu }^{e\left(p\right)}{â}_{\mu }^{†}{â}_{\nu }$$

Here, *Q* is the particle charge (−1 for electrons, +1 for protons, and, although not considered in this work, generally +*Z* for other quantized nuclei), and ${â}^{†}$ and â are fermionic creation and annihilation operators. The “e­(p)” notation indicates that the equation applies to both the electrons and the protons. The μ, ν, ζ, and η indices represent the electronic or protonic basis functions. The terms $d_{\mu \nu }^{e\left(p\right)}$ and $q_{\mu \nu }^{e\left(p\right)}$ are modified dipole and quadrupole integrals related to the standard electronic and protonic dipole and quadrupole integrals by

15a $$d_{\mu \nu }^{e\left(p\right)}=Q\underset{\alpha \in \left\{x,y,z\right\}}{\sum }\lambda_{\alpha }\left\langle \phi_{\mu }^{e\left(p\right)}\left|r_{\alpha }^{e\left(p\right)}\right|\phi_{\nu }^{e\left(p\right)}\right\rangle$$

15b $$q_{\mu \nu }^{e\left(p\right)}=Q\underset{\alpha ,\beta \in \left\{x,y,z\right\}}{\sum }\lambda_{\alpha }\lambda_{\beta }\left\langle \phi_{\mu }^{e\left(p\right)}\left|r_{\alpha }^{e\left(p\right)}r_{\beta }^{e\left(p\right)}\right|\phi_{\nu }^{e\left(p\right)}\right\rangle$$

where again *Q* is the particle charge, λ_{*x*,*y*,*z*}_ is a Cartesian component of the light–matter coupling vector **λ**, and *r*_{*x*,*y*,*z*}_ is a Cartesian component of the position vector **r**.

The third term of eq 13 is a joint electron–proton contribution to the self-dipole term. Notably, this term is formally an ab initio treatment of the self-dipole term for electrons coupled to quantum nuclei, which, to the best of our knowledge, has not been addressed in postfacto treatments of classical nuclei on conventional (non-NEO) QED potential energy surfaces. It is written in second quantization as

16 $$\left(\lambda \cdot {\mathrm{\mu ̂}}_{e}\right)\left(\lambda \cdot {\mathrm{\mu ̂}}_{p}\right)=\underset{\mu \nu \zeta \eta }{\sum }d_{\mu \nu }^{e}d_{\zeta \eta }^{p}{â}_{\mu }^{†}{â}_{\nu }{â}_{\zeta }^{†}{â}_{\eta }$$

where the μ and ν indices represent electronic basis functions and the ζ and η indices represent protonic basis functions.

Within the NEO Born–Oppenheimer approximation for classical nuclei, the fourth and fifth terms of eq 13 are one-body electronic and one-body protonic operators, respectively, scaled by a contribution from the classical nuclear dipole moment. These terms are written in second quantization as

17 $$\left(\lambda \cdot {\mathrm{\mu ̂}}_{e\left(p\right)}\right)\left(\lambda \cdot \mu_{N}\right)=d_{N}\underset{\mu \nu }{\sum }d_{\mu \nu }^{e\left(p\right)}{â}_{\mu }^{†}{â}_{\nu }$$

where

18 $$d_{N}=\lambda \cdot \mu_{N}$$

The final term in eq 13 is a scalar contribution to the total energy within the NEO Born–Oppenheimer approximation for classical nuclei.

For PN-QED-NEO-HF, the self-dipole electronic and protonic terms of eq 13 are included in the respective electronic and protonic Fock matrices. The one-body terms (i.e., the second term of eq 14 as well as eq 17) can be directly added to the Fock matrices. The many-body components of the self-dipole terms (i.e., the first term of eq 14 as well as eq 16) can be handled by folding the modified dipole integrals into the standard two-body integrals. We define the same-particle dipole-dressed integrals, ${\left(\mu \nu |\zeta \eta \right)}_{\mathrm{dd}}^{e\left(p\right)}$ , as

19 $${\left(\mu \nu |\zeta \eta \right)}_{\mathrm{dd}}^{e\left(p\right)}={\left(\mu \nu |\zeta \eta \right)}^{e\left(p\right)}+d_{\mu \nu }^{e\left(p\right)}d_{\zeta \eta }^{e\left(p\right)}$$

where (μν|ζη)^e(p)^ are the standard electron–electron (proton–proton) two-body integrals. Similarly, the mixed-particle dipole-dressed integrals are defined as

20 $${\left(\mu \nu |\zeta \eta \right)}_{\mathrm{dd}}^{\mathrm{ep}}={\left(\mu \nu |\zeta \eta \right)}^{\mathrm{ep}}+d_{\mu \nu }^{e}d_{\zeta \eta }^{p}$$

where (μν|ζη)^ep^ are the standard electron–proton two-body integrals with μ, ν representing electronic basis functions and ζ, η representing protonic basis functions. Utilizing the dipole-dressed integrals, the same-particle many-body self-dipole electronic and protonic terms (the first term of eq 14) are directly included in the standard Coulomb and exchange matrices of HF theory, while the mixed-particle self-dipole term (eq 16) is included as part of the electron–proton Coulomb matrix of NEO-HF theory.

For CS-QED-NEO-HF, the self-dipole term is expanded as

$$\begin{array}{rl} \frac{1}{2}{\left[\lambda \cdot \left(\mathrm{\mu ̂}-\left\langle \mathrm{\mu ̂}\right\rangle \right)\right]}^{2}= & \frac{1}{2}{\left(\lambda \cdot {\mathrm{\mu ̂}}_{e}\right)}^{2}+\frac{1}{2}{\left(\lambda \cdot {\mathrm{\mu ̂}}_{p}\right)}^{2}+\left(\lambda \cdot {\mathrm{\mu ̂}}_{e}\right)\left(\lambda \cdot {\mathrm{\mu ̂}}_{p}\right)\\ & -\left(\lambda \cdot {\mathrm{\mu ̂}}_{e}\right)\left(\lambda \cdot \left\langle {\mathrm{\mu ̂}}_{Q}\right\rangle \right)-\left(\lambda \cdot {\mathrm{\mu ̂}}_{p}\right)\left(\lambda \cdot \left\langle {\mathrm{\mu ̂}}_{Q}\right\rangle \right)\\ & +\frac{1}{2}{\left(\lambda \cdot \left\langle {\mathrm{\mu ̂}}_{Q}\right\rangle \right)}^{2} \end{array}$$ 21

where ${\mathrm{\mu ̂}}_{Q}$ is the dipole operator for only quantum particles, defined as ${\mathrm{\mu ̂}}_{Q}\equiv {\mathrm{\mu ̂}}_{e}+{\mathrm{\mu ̂}}_{p}$ . The first three terms of eq 21 are identical to those in eq 13 and are added to the electronic and protonic Fock matrices analogously. The next two terms are one-body electronic and one-body protonic dipole operators scaled by the quantum dipole operator expectation value for the current iteration. These one-body terms are added to the respective Fock matrices. The final term is a scalar contribution added to the total energy.

### 2.4 QED-NEO-CASCI

As stated in the Introduction, a general correlated QED-NEO-CI wave function has the form given by eq 3. In this work, the set of electronic (protonic) determinants included in our wave functions are complete active spaces considering all possible occupations of $N^{e\left(p\right)}$ electrons (protons) in $M^{e\left(p\right)}$ electronic (protonic) orbitals. The electronic and protonic orbitals for these active spaces can be obtained from a QED-NEO-HF calculation or from a bare (i.e., non-QED) NEO-HF calculation. The photon wave function is truncated to some maximum number of photons, $N_{\max}^{\mathrm{ph}}$ . We will denote these wave functions as $\left(N^{e},M^{e}\right)$ - $\left(N^{p},M^{p}\right)$ - $N_{\max}^{\mathrm{ph}}$ . The full wave function contains $N_{\det}^{\mathrm{tot}}=N_{\det}^{e}\;\times \;N_{\det}^{p}\;\times \;(N_{\max}^{\mathrm{ph}}+1)$ total coefficients, where $N_{\det}^{\mathrm{e(p)}}$ is the total number of electronic (protonic) determinants. For convenience, we define $N_{\det}^{\mathrm{NEO}}=N_{\det}^{e}\;\times \;N_{\det}^{p}$ as the number of products of determinants in the underlying NEO-CASCI wave function.

The wave function coefficients, *c*_*ijn*_, are obtained by diagonalization of the PF Hamiltonian. Here, we provide the expressions that allow the practical implementation of the QED-NEO-CASCI Hamiltonian. We first combine the molecular Hamiltonian with the self-dipole term to define the dipole-dressed molecular Hamiltonian,

22 $${Ĥ}_{\mathrm{mol}}^{\prime }={Ĥ}_{\mathrm{mol}}+\frac{1}{2}{\left(\lambda \cdot \mathrm{\mu ̂}\right)}^{2}$$

We introduce the shift operator for electrons,^59^ ${Ê}_{pq}$ , defined as

23 $${Ê}_{pq}=\underset{\sigma }{\sum }{â}_{p,\sigma }^{†}{â}_{q,\sigma }$$

where σ ∈ (α, β) is a spin index and *p*, *q* are indices that represent electronic molecular orbitals (distinct from the Greek indices for basis functions in Section 2.3). For protons, the shift operator ${Ê}_{PQ}$ is defined similarly,

24 $${Ê}_{PQ}={â}_{P}^{†}{â}_{Q}$$

Here, we use capital indices to represent protonic orbitals, and there is no summation over spin, as we assume a high-spin protonic wave function.

The dipole-dressed molecular Hamiltonian (eq 22) can be rewritten in terms of these shift operators as

Ĥmol′=∑pqhpqe′Êpq+12∑pqrs(pq|rs)dde(ÊpqÊrs−δqrÊps)+∑PQhPQp′ÊPQ+12∑PQRS(PQ|RS)ddp(ÊPQÊRS−δQRÊPS)+∑pqPQ(pq|PQ)ddepÊpqÊPQ+Ediag 25

Here, *E*_diag_ is a scalar energy contribution resulting from the self-dipole term, $h_{pq}^{e^{\prime }}$ is a modified electronic core Hamiltonian, ${\left(pq|rs\right)}_{\mathrm{dd}}^{e}$ are the dipole-dressed electronic two-body integrals of eq 19 transformed into the molecular orbital basis, $h_{PQ}^{p^{\prime }}$ and ${\left(PQ|RS\right)}_{\mathrm{dd}}^{p}$ are defined analogously for protons, and ${\left(pq|PQ\right)}_{\mathrm{dd}}^{\mathrm{ep}}$ are the mixed-particle dipole-dressed integrals of eq 20 transformed to the molecular orbital basis.

The forms of the modified core Hamiltonians and diagonal energy contribution depend on the representation of the cavity photon. Akin to QED-CASCI,^35^ both the photon number and coherent state representations can be used within QED-NEO-CASCI. In this work, we choose to work exclusively in the photon number representation, as it gives a clear interpretation of the photon wave function and allows us to avoid approximating the quantum dipole operator expectation value. The expressions for the coherent state representation and further rationale for this choice are provided in the . In the photon number representation, the modified electronic and protonic core Hamiltonians are given by

26a $$h_{pq}^{e^{\prime }}=h_{pq}^{e}-\frac{1}{2}q_{pq}^{e}+d_{N}d_{pq}^{e}$$

26b $$h_{PQ}^{p^{\prime }}=h_{PQ}^{p}+\frac{1}{2}q_{PQ}^{p}+d_{N}d_{PQ}^{p}$$

Here, $h_{pq}^{e}$ $\left(h_{PQ}^{p}\right)$ is the standard electronic (protonic) core Hamiltonian in the molecular orbital basis, $d_{pq}^{e}$ $\left(d_{PQ}^{p}\right)$ and $q_{pq}^{e}$ $\left(q_{PQ}^{p}\right)$ are the modified electronic (protonic) dipole and quadrupole integrals of eq 15a and eq 15b, respectively, transformed into the molecular orbital basis, and *d*_*N*_ is the dipole contribution from the classical nuclei defined in eq 18. The diagonal energy contribution is

27 $$E_{\mathrm{diag}}=\frac{1}{2}d_{N}^{2}$$

The bilinear coupling in the photon number representation is expressed as

28 $${Ĥ}_{\mathrm{blc}}=-\sqrt{\frac{\omega }{2}}\left(\underset{pq}{\sum }d_{pq}^{e}{Ê}_{pq}+\underset{PQ}{\sum }d_{PQ}^{p}{Ê}_{PQ}+d_{N}\right)\left(b^{†}+b\right)$$

Note that in all the expressions in this section, we have used general indices *p*, *q*, *r*, *s* for electrons and *P*, *Q*, *R*, *S* for protons. It is common in NEO-CASCI calculations to have doubly occupied inactive electronic orbitals in addition to the active orbitals, in which case the electronic summations can be split into summations over inactive and active orbitals.

A schematic of the total QED-NEO-CASCI Hamiltonian is shown in Figure 1. Blocked on the photon number basis states, the Hamiltonian is tridiagonal because the bilinear coupling only contains operators that couple blocks of ±1 photon. The blocks diagonal in photon number contain replicas of the dipole-dressed molecular Hamiltonian along with the diagonal addition of the bare cavity energy, ω*b* ^†^ *b*. As such, the molecular Hamiltonian sub-block only needs to be constructed once and can then be copied into the remaining diagonal blocks with the bare cavity energy then added along the diagonal. From a computational perspective, the most expensive components of the full Hamiltonian are the many-body electron–electron, proton–proton, and electron–proton operators in eq 25, so only constructing this sub-block of the Hamiltonian once is optimal. Moreover, because the self-dipole term is entirely folded into the molecular Hamiltonian, this sub-block construction can be accomplished with reuse of existing NEO-CASCI code.

**Figure 1:**
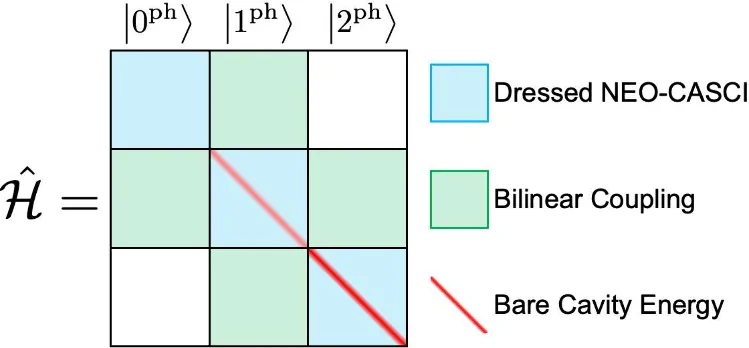
Schematic representation of the QED-NEO-CASCI Hamiltonian, shown blockwise with respect to photon number basis states. The full matrix is of dimension $N_{\det}^{\mathrm{tot}}\;\times \;N_{\det}^{\mathrm{tot}}$ , where each of the blocks is of the dimensionality $N_{\det}^{\mathrm{NEO}}\;\times \;N_{\det}^{\mathrm{NEO}}$ . Plain white blocks are zero, and the red diagonal line indicates the addition of the bare cavity energy contribution.

The different bilinear coupling blocks in the Hamiltonian differ only by the coefficients resulting from the bosonic photon raising and lowering operators. Similar to the diagonal blocks, these blocks only require one construction of the molecular component of the bilinear coupling, $-\sqrt{\frac{\omega }{2}}\left(\underset{pq}{\sum }d_{pq}^{e}{Ê}_{pq}+\underset{PQ}{\sum }d_{PQ}^{p}{Ê}_{PQ}+d_{N}\right)$ . This molecular component is then scaled by the factors resulting from the action of *b* and *b* ^†^ (e.g., $\sqrt{n}$ from $b\left|n\right\rangle =\sqrt{n}\left|n-1\right\rangle$ and $\sqrt{n+1}$ from $b^{†}\left|n\right\rangle =\sqrt{n+1}\left|n+1\right\rangle$ ) before being added to the respective off-diagonal blocks of the total Hamiltonian.

For practical calculations, the full matrix is not constructed, and instead direct diagonalization algorithms are used. These algorithms only require building the vector σ⃗ resulting from the matrix-vector product of the Hamiltonian action on a trial vector,^60^ σ⃗=Ĥ∗C⃗ . This direct matrix-vector product is computed by sequentially applying the terms within the full Hamiltonian to the trial vector, as shown schematically in Figure 2. The bare cavity energy is trivial to apply, as it is diagonal for each photon block of the total trial vector. To apply the dipole-dressed molecular Hamiltonian, we recast the trial vector from a single vector of size $N_{\det}^{\mathrm{tot}}$ to $\left(N_{\max}^{\mathrm{ph}}+1\right)$ vectors of size $N_{\det}^{\mathrm{NEO}}$ . This recasting reduces the problem to a multiroot NEO-CASCI matrix-vector multiplication, at which point existing direct NEO-CASCI code can be reused. The final term, the bilinear coupling, couples the different photon number blocks. This term contains only one-body electronic and protonic operators and therefore is not expensive to compute directly.

**Figure 2:**
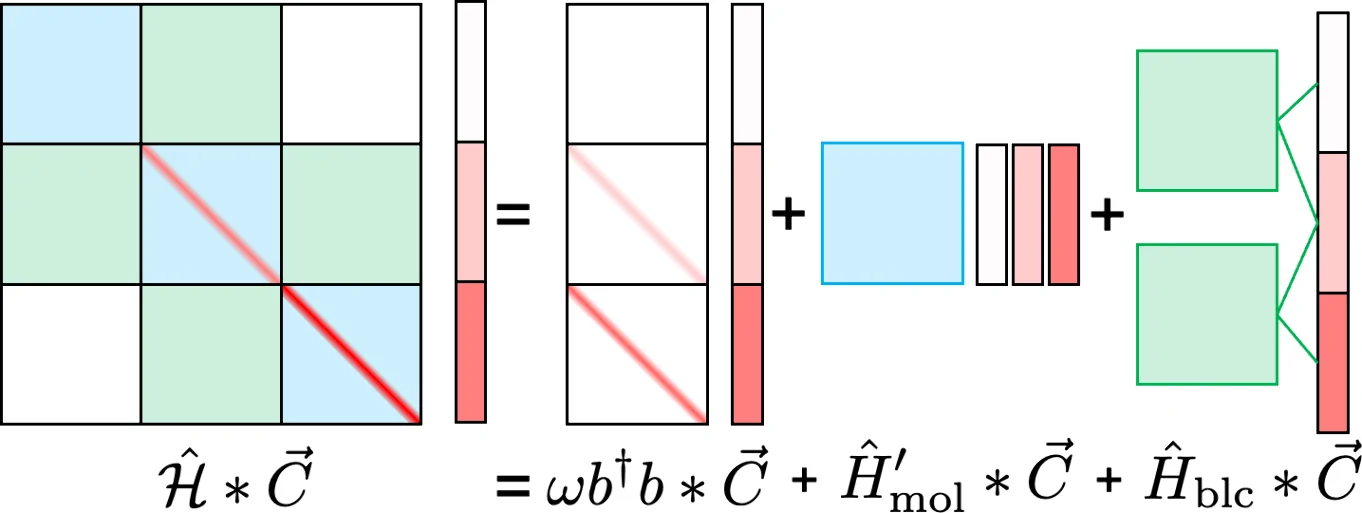
Schematic representation of the direct matrix-vector product of the full QED-NEO-CASCI Hamiltonian on a trial vector, C⃗ . The full Hamiltonian is identical to that in Figure 1. All vectors and matrices are depicted in the same photon number basis and use the same color scheme as Figure 1. The trial vector is of dimension $N_{\det}^{\mathrm{tot}}$ .

### 2.5 QED-NEO-TDCI

To simulate the dynamics of a system with electrons, quantized nuclei, and photons, including all nonadiabatic couplings among them, we use a real-time dynamics approach. In QED-NEO-TDCI, the wave function becomes

29 $$\left|\Psi \left(t\right)\right\rangle =\underset{ijn}{\sum }c_{ijn}\left(t\right)\left|\Phi_{i}^{e}\right\rangle \left|\Phi_{j}^{p}\right\rangle \left|n^{\mathrm{ph}}\right\rangle$$

where all time dependence is carried by the wave function coefficients. The orbitals that comprise the electronic and protonic determinants are fixed as the orbitals obtained from a preceding QED-NEO-HF or NEO-HF calculation.

We propagate this time-dependent wave function using a symplectic split operator, which has been used previously in both conventional CASCI^61^ and NEO-CASCI^50^ contexts. Briefly, the time-dependent wave function coefficients, *c*_*ijn*_(*t*), are split into real and imaginary components. The propagator, $e^{-iĤ\left(t\right)t}$ , contains a time-dependent Hamiltonian, which is the sum of the time-independent PF Hamiltonian and any time-dependent perturbations, such as applied external fields. The propagator is linearized to numerically propagate the wave function forward in time.^61^

## 3 Computational Details

We have implemented QED-HF, QED-CASCI, QED-NEO-HF, and QED-NEO-CASCI in both the photon number and coherent state photon representations. All methods have been implemented in a development version of the Chronus Quantum software package.^62^ Our implementations have been validated, where possible, against available open-source QED-HF^13,28^ and QED-CASCI^35^ implementations. While not the focus of this work, we provide sample QED-NEO-HF calculations in the . These calculations demonstrate that even at the mean-field level, quantized nuclear wave functions can be altered in QED environments.

The first system used to highlight the capabilities of QED-NEO-CASCI is the HeHHe^+^ molecular cation. At large He–He distances in a conventional Born–Oppenheimer picture, the central proton experiences a double-well potential.^63^ The two lowest-energy vibrational wave functions are the symmetric and antisymmetric linear combinations of vibrational wave functions in each of these wells, and the energy difference is called the tunneling splitting. We will refer to the symmetric and antisymmetric states as $\left|\Psi_{S}\right\rangle$ and $\left|\Psi_{A}\right\rangle$ , respectively. Within the NEO framework, multireference treatments are required to even qualitatively correctly describe these bilobal protonic wave functions.^63,64^ We used a He–He distance of 2.2 Å, with the He atoms placed symmetrically about the origin along the *z*-axis. The proton basis function centers and associated electronic basis function centers were placed symmetrically 0.28 Å away from the origin along the *z*-axis. The center of charge is therefore at the origin for all calculations. We used the 6-31G^65^ electronic and PB4-D^66^ protonic basis sets, which are small but chosen to allow for full CI calculations. For all HeHHe^+^ calculations, we used a 4-electron 8-orbital electronic active space, which, for this model system, is equivalent to a full CI calculation, and a 1-proton 18-orbital protonic active space, containing only the orbitals polarized along the *z*-axis. Our electronic and nuclear orbitals were taken from a NEO-HF calculation, and we worked in the photon number representation for QED-NEO-CASCI.

For all calculations, the cavity mode was polarized along the *z*-axis, and the reported light–matter coupling strength is the scalar strength along this axis. We used a maximum of $N_{\max}^{\mathrm{ph}}$ = 4 photons, for a final wave function definition of (4e,8o)-(1p,18o)-4. For the VSC and vibronic strong coupling studies in Sections 4.1 and 4.3, respectively, calculations performed at the largest light–matter coupling strength using a maximum of 9 photons produced the same absolute energies to within 2 × 10^–7^ Hartree, confirming that using a maximum of 4 photons is sufficiently converged. For the QED-NEO-TDCI tunneling dynamics calculations, a time-step of 0.01 a.u. was used, and the wave functions were propagated for a total of 16 000 a.u. (∼387 fs). The photon number state populations were saved every 50 timesteps, while the one-dimensional slices of the electronic and nuclear densities were generated every 2000 timesteps (∼0.48 fs).

The second system used to highlight the capabilities of QED-NEO-CASCI is a water dimer, which has been shown to undergo geometric changes with increasing light–matter coupling strength at the QED-HF level of theory.^67^ We optimized a water dimer geometry at the conventional and bare (i.e., non-NEO and non-QED) MP2/cc-pVDZ^68^ level theory in Q-Chem 6.4.^69^ To simplify our analysis, we applied a constraint ensuring that the O–H–O atoms involved in the hydrogen bond remain colinear along the *z*-axis. The geometry in Cartesian coordinates is provided in the . The cc-pVTZ^68^ electronic and PB5-F^66^ protonic basis sets were used for the QED-NEO-CASCI calculations. The electronic active space was (6e,6o), with orbitals chosen from a NEO-MP2 ^70^ natural orbital calculation. These orbitals were chosen to be polarized along the hydrogen bond. Plots of these electronic orbitals are provided in the . The protonic active space was (1p,46o), which includes all protonic orbitals.

The cavity mode was polarized along the hydrogen bond (i.e., the *z*-axis), and again the light–matter coupling strengths are reported as the scalar coupling strength along the *z*-axis. We used the photon number representation with a maximum of 6 photons, for a total wave function of (6e,6o)-(1p,46o)-6. Calculations performed at the largest light–matter coupling strength using a maximum of 9 photons produced the same absolute energies to within 2 × 10^–7^ Hartree, confirming that using a maximum of 6 photons is sufficiently converged.

Throughout this work, we use light–matter coupling strengths from 0.001 a.u. to 0.1 a.u. Our largest light–matter coupling strength, 0.1 a.u., corresponds to a cavity volume of ∼0.19 nm^3^, which may be experimentally realizable in plasmonic nanocavity environments,^71^ where cavity geometries with volumes as low as 0.15 nm^3^ have been designed.^72^ VSC can also be achieved in Fabry–Perot cavities with volumes as large as microliters containing a large number of molecules.^73^ In this case, our light−matter coupling strengths may be viewed as the coupling to an ensemble of dipole-aligned molecules, as is common in the QED literature.^14^

## 4 Results and Discussion

### 4.1 Vibrational Strong Coupling to Tunneling States in HeHHe^+^

We first demonstrate QED-NEO-CASCI by coupling the cavity mode to the proton tunneling vibrational excitation of HeHHe^+^. The cavity frequency was set to be resonant with the tunneling splitting outside the cavity, which for the geometry, basis sets, and active spaces used herein is 552 cm^–1^. A schematic representation of the coupling to form polaritonic states under VSC is shown in Figure 3. Before presenting numerical results, we frame our discussion around this simple model. In QED-NEO-CASCI, if the bilinear coupling is turned off $\left({Ĥ}_{\mathrm{blc}}=0\right)$ , diagonalizing our PF Hamiltonian gives $N_{\max}^{\mathrm{ph}}+1$ blocks of states, $\left|\left\{\Psi_{m}^{\prime }\right\},0\right\rangle ,\left|\left\{\Psi_{m}^{\prime }\right\},1\right\rangle , ...\left|\left\{\Psi_{m}^{\prime }\right\},N_{\max}^{\mathrm{ph}}\right\rangle$ . Here, $\left\{\Psi_{m}^{\prime }\right\}$ is the set of vibronic states resulting from diagonalizing only the dipole-dressed molecular Hamiltonian ( ${Ĥ}_{\mathrm{mol}}^{\prime }$ of eq 25), and the second index is the number of photons. These blocks differ by only a vertical energy offset of *n*ω, where *n* is the number of photons. We will thus refer to these states as “molecule plus photon states” because they have a clear interpretation as a cavity-free molecular state plus an integer number of photons. When the bilinear coupling is turned on, blocks differing by ±1 photon are coupled, resulting in the polaritonic states. Access to these full polaritonic states through diagonalization of the PF Hamiltonian allows us to extract the contributions of each molecule plus photon state to the lower polaritonic (LP) and upper polaritonic (UP) wave functions.

**Figure 3:**
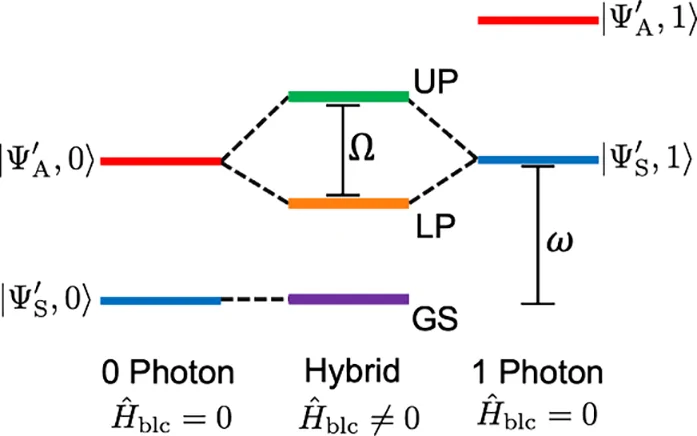
Schematic of the relevant vibrational states involved in VSC with fundamental cavity frequency ω. The molecular states with no photon contribution (0 photon column) are hybridized with the same states dressed with one cavity photon (1 photon column) to create the hybrid light–matter states, called the lower polaritonic (LP) and upper polaritonic (UP) states with Rabi splitting frequency Ω. The ground state (GS) remains the same in this picture.

To define the molecular components of the molecule plus photon states, we can use calculations performed outside the cavity (e.g., NEO-CASCI calculations). Notably, these states are eigenfunctions of the molecular Hamiltonian, ${Ĥ}_{\mathrm{mol}}$ , as opposed to the dipole-dressed Hamiltonian, ${Ĥ}_{\mathrm{mol}}^{\prime }$ . These calculations use the same electronic and protonic active spaces as the corresponding QED-NEO-CASCI calculations. We will use $\left\{\Psi_{m}^{\prime }\right\}$ to denote eigenfunctions of ${Ĥ}_{\mathrm{mol}}^{\prime }$ and {Ψ_*m*_} to denote the analogous eigenfunctions of ${Ĥ}_{\mathrm{mol}}$ . For sufficiently small light–matter coupling strengths, as used in this work, these sets of eigenstates are qualitatively indistinguishable (see ) and thus can be used interchangeably to interpret QED-NEO-CASCI wave functions.

Two features of the simple model in Figure 3 are important for interpreting our results. First, the polaritonic states are formed by combining molecule plus photon states comprised of the same manifold of vibronic states with different numbers of photons. The LP and UP states in Figure 3, for example, are formed by mixing a state best described as the first vibronic excited state with no photons, $\left|\Psi_{A}^{\prime },0\right\rangle$ , and the vibronic ground state with one photon, $\left|\Psi_{S}^{\prime },1\right\rangle$ . Second, by providing a fully ab initio description of all vibronic states, we enable the possibility of nonresonant coupling. For example, as the light–matter coupling strength is increased, the UP state may begin to strongly mix with $\left|\Psi_{A}^{\prime },1\right\rangle$ as these states become nearly degenerate. The second feature highlights the insights offered by QED-NEO-CASCI beyond what is offered using simple physical models, such as the Jaynes–Cummings Hamiltonian.^74^

Returning to HeHHe^+^, the energies of the LP and UP states relative to the ground state (GS) as a function of light–matter coupling strength are shown in Figure 4. For reference, the absolute energies of the ground state as well as the LP and UP states as a function of light–matter coupling strength are shown in . We observe linear scaling of the Rabi splitting with the light–matter coupling strength. This linear scaling is in agreement with the results from model Hamiltonians^10^ and confirms the ability of QED-NEO-CASCI to provide an ab initio description of VSC.

**Figure 4:**
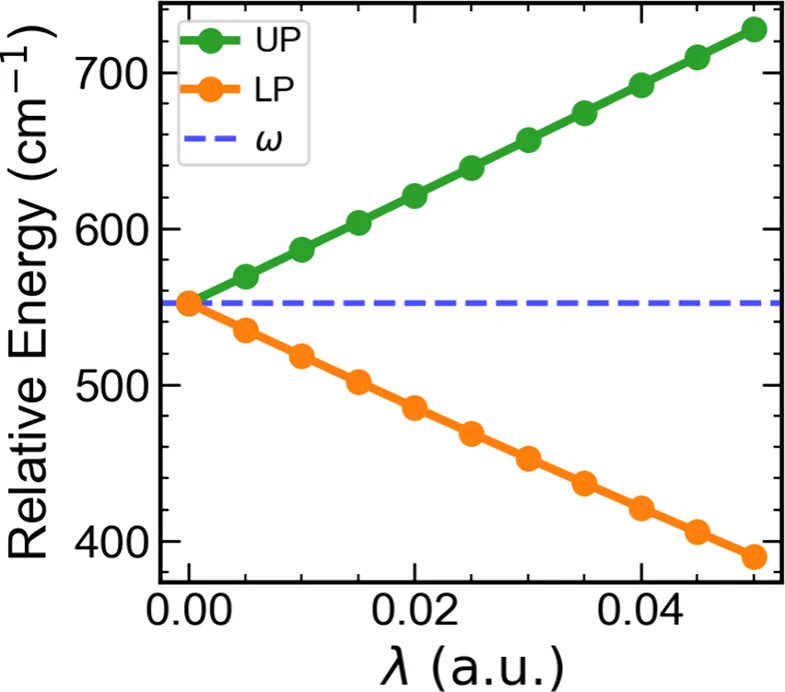
QED-NEO-CASCI energies of the LP and UP states relative to the GS for HeHHe^+^ coupled to a cavity mode resonant with the tunneling splitting with light–matter coupling strength λ. The dashed blue line indicates the cavity frequency. The difference between the solid green and orange lines is the Rabi splitting and scales linearly with λ.

Next we analyze the polaritonic wave functions. For this purpose, we perform a natural orbital transformation^75^ for each of the GS, LP state, and UP state by diagonalizing the electronic and protonic one-body reduced density matrices. Figure 5 shows the proton orbitals and photon populations of the most important molecule plus photon states for each of these states at a light–matter coupling strength of λ = 0.01 a.u. For all molecule plus photon states relevant to Figure 5, the electronic determinant is the Aufbau determinant. This analysis is only possible by having access to the time-independent QED-NEO-CASCI wave functions and thus could not be performed using time-dependent semiclassical treatments of the cavity mode.^54^

**Figure 5:**
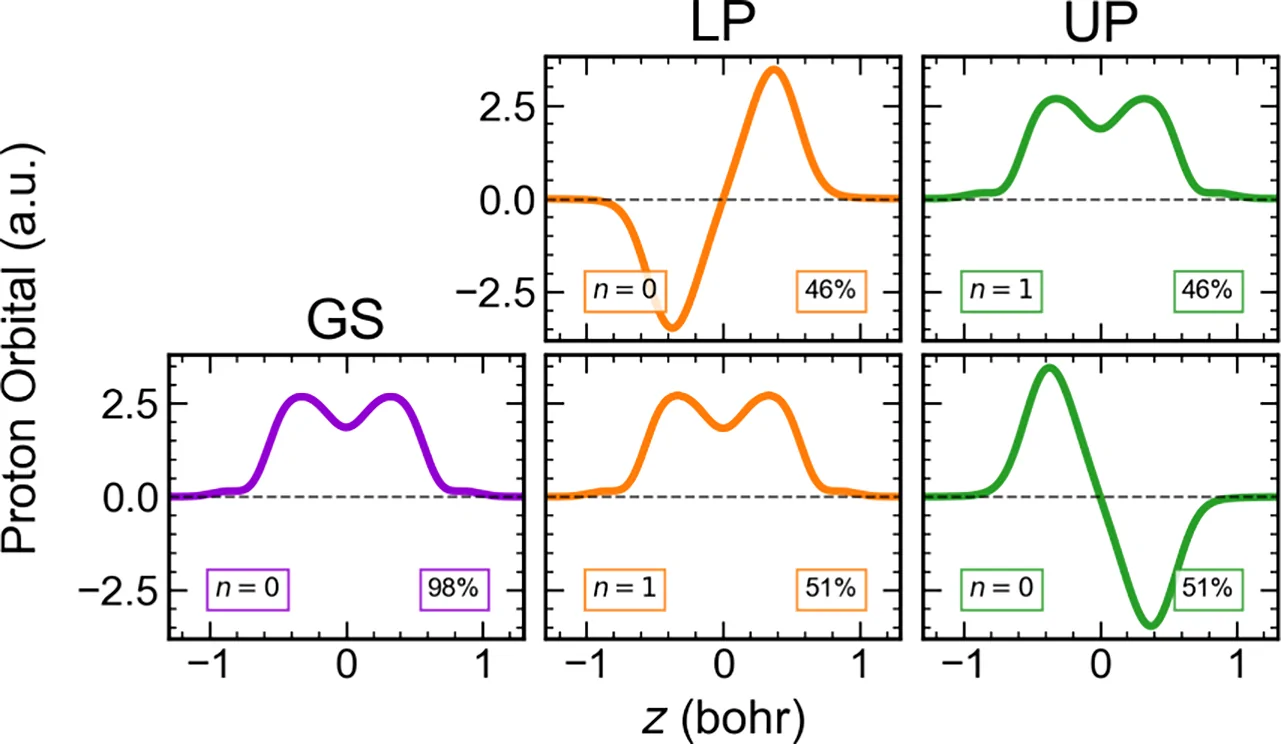
Proton orbitals and photon occupation numbers for the most important molecule plus photon states involved in the GS, LP state, and UP state for HeHHe^+^ coupled to a cavity mode resonant with the tunneling splitting using a light–matter coupling strength of λ = 0.01 a.u. Each plot corresponds to one molecule plus photon state and includes a one-dimensional slice of the proton density along the *z*-axis, the number of photons in the lower left corner, and the overall contribution of that state to the total wave function in the lower right corner. An orbital with no nodes is $\left|\Psi_{S}^{\prime }\right\rangle$ , and an orbital with one node is $\left|\Psi_{A}^{\prime }\right\rangle$ . Only molecule plus photon states comprising >1% of the total wave function are included.

As expected, the ground state is primarily (98%) composed of $\left|\Psi_{S}^{\prime },0\right\rangle$ . The remaining 2% of the ground state is mainly composed of contributions from other states associated with electron–electron and electron–proton correlation, although there is some coupling to states containing nonzero photon occupations, most notably $\left|\Psi_{A}^{\prime },1\right\rangle$ . The LP and UP states are each primarily composed of two molecule plus photon states, $\left|\Psi_{S}^{\prime },1\right\rangle$ and $\left|\Psi_{A}^{\prime },0\right\rangle$ . In a minimal coupling model, these states are combined into the maximally entangled Bell states given by^76^

30 $$\left|\Phi_{\pm }\right\rangle =\frac{1}{\sqrt{2}}\left(\left|\Psi_{S}^{\prime },1\right\rangle \pm \left|\Psi_{A}^{\prime },0\right\rangle \right)$$

In the language of quantum chemistry, the entanglement is an indication that the polaritonic states include strong (static) correlation between the vibrational and photonic degrees of freedom. However, because of the full ab initio description provided by QED-NEO-CASCI, our states have more complexity than the minimal coupling model. Specifically, the LP and UP states are not composed of equal weights of these molecule plus photon states; one molecule plus photon state is 51% and the other is 46% of the polaritonic state. At this relatively small light–matter coupling strength, there is no straightforward way to distinguish between coupling to states associated with electron–electron and electron–proton correlation versus coupling to other photon occupations. However, at larger values of light–matter coupling, there are noticeable contributions from higher occupied photon number states. At the largest light–matter coupling strength considered, λ = 0.05 a.u., the ground state gains a 2% contribution from $\left|\Psi_{A}^{\prime },1\right\rangle$ , and the two polaritonic states each gain a 2% contribution from $\left|\Psi_{A}^{\prime },2\right\rangle$ .

To obtain further insight, although a physical cavity will couple to all electrons and protons, with our QED-NEO-CASCI code we are able to selectively couple to solely either electrons or protons by coupling the cavity mode to only the respective particle’s dipole operator. This analysis provides insight into the quantum mechanical coupling between quantized particles. For this tunneling system, the LP and UP energies calculated when coupling only to either the electrons or the proton are shown in Figure 6. For notational convenience, we will denote the Rabi splitting arising when the cavity mode is coupled only to the electrons or only to the proton as Ω_e_ and Ω_p_, respectively, and the Rabi splitting when simultaneously coupling the cavity mode to both the electrons and the proton as Ω_ep_. Both Ω_e_ and Ω_p_ are less than Ω_ep_. At small light–matter couplings, Ω_ep_ – (Ω_e_ + Ω_p_) ≈ 0, but this quantity becomes more negative as the light–matter coupling strength increases, as shown in . In the future, it will be interesting to utilize QED-NEO-CASCI to study how coupling a cavity mode to different molecular degrees of freedom impacts VSC, particularly in the ultrastrong coupling regime, where the self-dipole electronic–nuclear term (eq 16) specific to our QED-NEO treatment may become important.

**Figure 6:**
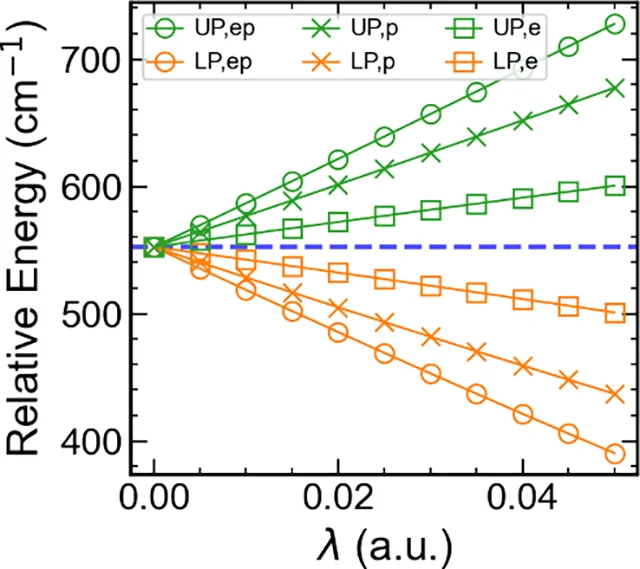
QED-NEO-CASCI energies of the LP and UP states for HeHHe^+^ when the cavity is coupled to both the electrons and the proton (ep), solely to electrons (e), or solely to the proton (p) as a function of light–matter coupling strength λ. The dashed blue line indicates the cavity frequency. The difference between pairs of green and orange lines coupled to the same subsets of particles is the Rabi splitting for that coupling scheme.

### 4.2 Hydrogen Tunneling Dynamics in HeHHe^+^

Next, we used QED-NEO-TDCI to simulate the real-time nonequilibrium, nonadiabatic dynamics of hydrogen tunneling in HeHHe^+^ by constructing an initial wave function that approximately localizes the proton near one of the two helium atoms (i.e., in one of the two wells of the double-well potential in the conventional framework). We have previously simulated these dynamics both outside the cavity^50^ and when coupling the molecule to a classical cavity mode.^54^ In the latter simulations, we observed the cavity modification of the hydrogen tunneling dynamics. With QED-NEO-TDCI, the photon mode and its interactions with the electrons and quantized proton are properly treated fully quantum mechanically, which has been shown to include additional physics beyond the mean-field description of the classical cavity mode.^52^

Outside a cavity, the quantized proton is approximately localized near one of the helium atoms by choosing an initial wave function of

31 $$\left|\Psi \left(t=0\right)\right\rangle =\frac{1}{\sqrt{2}}\left(\left|\Psi_{S}\right\rangle +\left|\Psi_{A}\right\rangle \right)$$

It can be shown analytically^50^ that the time-dependent total dipole moment for this system is proportional to cos­(Δ*Et*), where Δ*E* is the tunneling splitting energy. For our QED-NEO-TDCI simulations, we chose the initial wave function to be

32 $$\left|\Psi \left(t=0\right)\right\rangle =\frac{1}{\sqrt{2}}\left(\left|\Psi_{S},n\right\rangle +\left|\Psi_{A},n\right\rangle \right)$$

where *n* is either 0 or 1 (i.e., either no photons or one photon in the initial wave function). This wave function is constructed by taking the $\left|\Psi_{S}\right\rangle$ and $\left|\Psi_{A}\right\rangle$ wave functions from a cavity-free calculation and splicing them scaled by $\frac{1}{\sqrt{2}}$ into the respective photon number blocks of the total QED-NEO-CASCI wave function.

The time-dependent dipole moments and photon number state populations for the trajectories initialized according to eq 32 are shown in Figure 7. For the trajectories shown, we used a light–matter coupling strength of λ = 0.05 a.u. to clearly observe the cavity impact while propagating relatively short trajectories. Additional trajectories propagated with light–matter coupling strengths of λ = 0.01 a.u. and λ = 0.03 a.u. are provided in the . In order to observe the impact of coupling the molecule to the cavity on the dynamics, we also plot the trace of the time-dependent dipole moment for a cavity-free trajectory, which again oscillates as a simple cos­(Δ*Et*) function.

**Figure 7:**
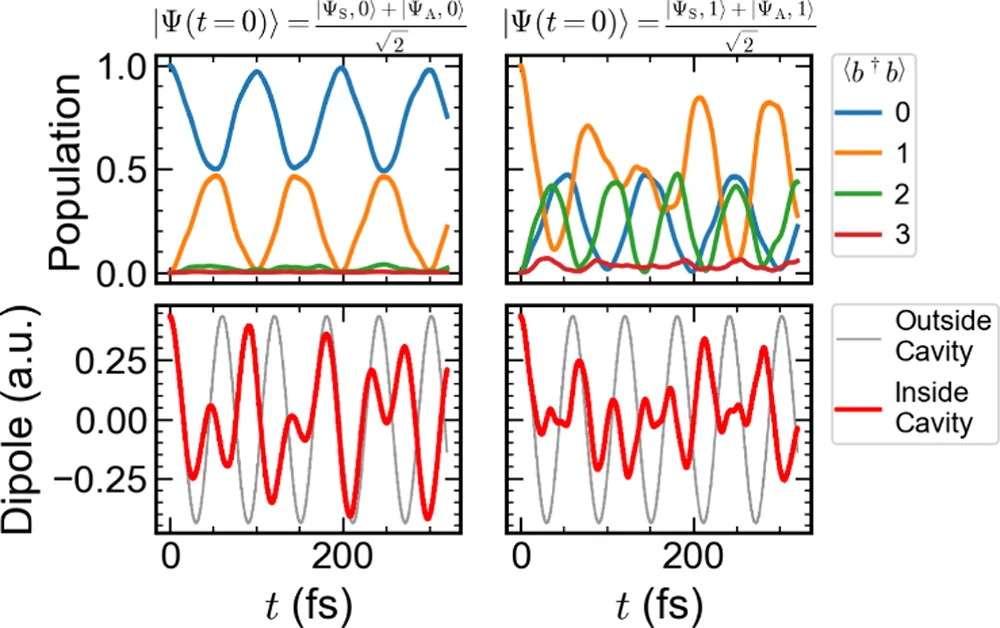
Time-dependent photon number state populations (top row) and molecular dipole moments (bottom row) for QED-NEO-TDCI trajectories of HeHHe^+^ initialized from the localized state given by eq 32 utilizing a light–matter coupling strength of λ = 0.05 a.u. Although the cavity can be populated by up to 4 photons, only the populations up to 3 photons are shown for clarity, as the 4 photon number state gains negligible population in all trajectories. For the molecular dipole moments, the dipole moment of a RT-NEO-CASCI trajectory initialized from eq 31 and propagated outside the cavity is shown in gray for reference.

Clearly, coupling to the cavity significantly impacts the hydrogen tunneling dynamics, as shown by the modified time-dependent dipole moments. Starting with the trajectory that initially contains no photons (left column of Figure 7), we observe energy exchange between the molecular and photon degrees of freedom. The trajectory starts with the proton localized near one He atom in a nonequilibrium state, as indicated by the nonzero molecular dipole moment. As the trajectory is propagated, energy is transferred from the molecule to the cavity, as indicated by the photon number state population oscillating between populating solely the 0-photon state and populating an approximately even mixture of the 0- and 1-photon states. Populating the 1-photon state temporally correlates with substantial deviations of the time-dependent dipole moment from the cavity-free trajectory at around 50, 150, and 250 fs. A small population of the 2-photon state arises around the same time the 1-photon state is populated. When energy is returned to the molecule, as indicated by the nearly unity population of the 0-photon state at around 100, 200, and 300 fs, the proton localizes near one of the He atoms, as indicated by the nearly maximum magnitudes of the dipole moments. This localization alternates between being in phase (i.e., localized near the same He atom) with the cavity-free trajectory, for example, around 200 fs, and out of phase (i.e., localized near different He atoms) with the cavity-free trajectory, for example, at around 100 and 300 fs. We also note that the Fourier transform of the time-dependent dipole moment contains two primary peaks that occur at roughly the excitation energies of the upper and lower polariton states for this system (). The Fourier transform also contains minor peaks at higher energies, although the physical origins of these peaks are unclear. This analysis shows how coupling to a cavity can alter the hydrogen tunneling dynamics.

When the wave function is initialized with one photon (right column of Figure 7), much more complicated dynamics occur, as reflected by the time-dependent dipole moment and its Fourier transform (), as well as the photon number state populations. As the initial wave function involves excitation of both the molecule and the cavity, a bidirectional flow of energy occurs. In this case, we observe population transfer from the 1-photon state to primarily the 0- and 2-photon states, although the 3-photon state also gains some population. The population of the 3-photon state arises from indirect coupling through the 2-photon state, which occurs when relatively large light–matter coupling is used. Indeed, if we use a small light–matter coupling of λ = 0.01 a.u., the 3-photon state does not gain significant population (see ).

In addition to the time-dependent photon number state populations, we plotted the time-dependent electronic and protonic densities. For the trajectories shown in Figure 7, the includes movies showing one-dimensional slices of both the electronic and protonic densities evolving with the changing photon number state populations. The also includes a movie showing the electronic and protonic densities outside the cavity for reference. These movies illustrate the interplay among the proton, electrons, and photons as energy is exchanged between the molecular and cavity degrees of freedom. These results highlight the nonequilibrium, nonadiabatic dynamics that can be captured by the QED-NEO-TDCI method.

### 4.3 HeHHe^+^ Vibronic Strong Coupling

We now couple the cavity mode to a vibronic progression associated with the *S*_0_ → *S*_1_ electronic transition. Within the NEO framework, vibronic progressions are doubly excited states with both electronic and nuclear (vibrational) excitations. Vibronic progressions are not accessible with lower-scaling NEO excited-state methods such as NEO-TDDFT,^77^ which is limited to describing single excitations or linear combinations of single excitations. To describe double excitations, we will specify the molecule plus photon states with the labels $\left|S_{i},\nu ,n\right\rangle$ , where *S*_*i*_ indicates the electronic state and will be either *S*_0_ or *S*_1_, ν indicates the number of quanta in the proton vibrational mode along the *z* axis, and *n* indicates the number of photons. To denote vibronic states outside the cavity, we omit the *n* index (e.g., $\left|S_{0},0\right\rangle$ is the ground state outside the cavity).

We couple the cavity mode to the $\left|S_{0},0\right\rangle \to \left|S_{1},0\right\rangle$ molecular transition, which corresponds to a cavity frequency of ω = 19.26 eV. A schematic depiction of this system is shown in Figure 8. To plot the spectra, we calculate the oscillator strength for each transition as

33 $$f\left(\Delta E\right)\propto \Delta E\underset{nn^{\prime }}{\sum }\;\underset{iji^{\prime }j^{\prime }}{\sum }c_{ijn}^{*}c_{i^{\prime }j^{\prime }n^{\prime }}|\left\langle \Phi_{i}^{e}\right|\left\langle \Phi_{j}^{p}\right|\mathrm{\mu ̂}\left|\Phi_{i^{\prime }}^{e}\right\rangle \left|\Phi_{j^{\prime }}^{p}\right\rangle {|}^{2}\delta_{n,n^{\prime }}$$

where Δ*E* is the transition energy from the ground state. HeHHe^+^ is a molecule with a high degree of symmetry, including the center of inversion symmetry. The *S*_0_ electronic state is symmetric with respect to inversion, while the *S*_1_ state is antisymmetric with respect to inversion. The vibrational states with an even number of excitation quanta (ν = 0, 2, 4, ...) are symmetric with respect to inversion, while vibrational states with an odd number of excitation quanta (ν = 1, 3, 5, ...) are antisymmetric with respect to inversion. Because the dipole operator is antisymmetric with respect to inversion, the only dipole allowed transitions in the $\left|S_{0},0\right\rangle \to \left|S_{1},\nu \right\rangle$ vibronic progression are those that have an even number of quanta in the vibrational mode on the electronic excited state (e.g., $\left|S_{1},0\right\rangle$ , $\left|S_{1},2\right\rangle ,\left|S_{1},4\right\rangle ,...$ ). These optically allowed transitions are indicated with arrows in the 0 photon block of Figure 8.

**Figure 8:**
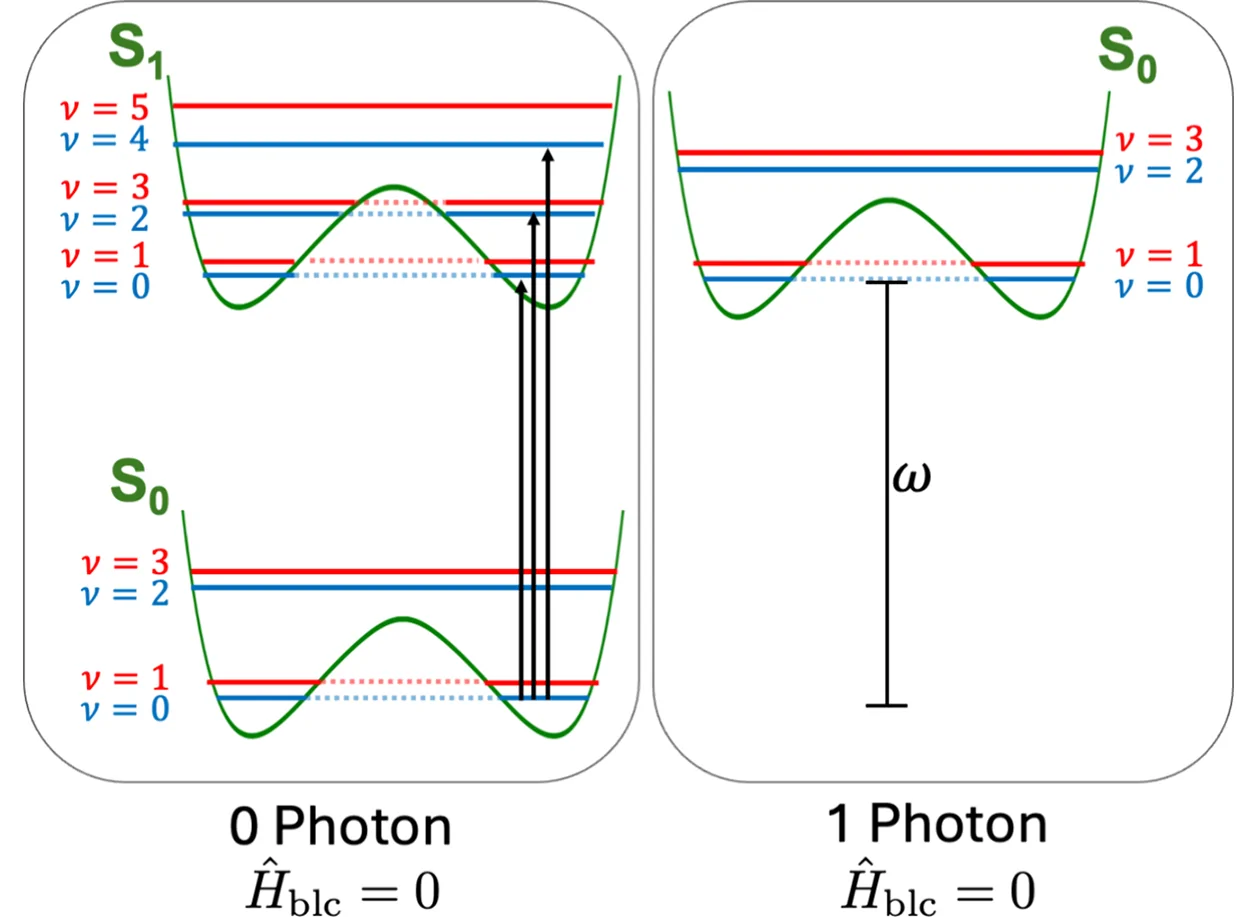
Schematic depiction of the 0- and 1-photon dressed states relevant for the vibronic coupling calculations. The dressed states do not include the bilinear coupling (i.e., ${Ĥ}_{\mathrm{blc}}=0$ ). The vertical arrows shown in the 0 photon block are optically allowed transitions of the vibronic progression outside the cavity. The cavity frequency is ω.

The spectra calculated at various light–matter coupling strengths are shown in Figure 9, along with the spectrum obtained outside the cavity for reference. As mentioned above, the primary molecular transition coupled to the cavity mode corresponds to $\left|S_{0},0\right\rangle \to \left|S_{1},0\right\rangle$ . Beginning with the smallest light–matter coupling, λ = 0.001 a.u., we observe a small splitting ( ≈0.023 eV) of the primary transition into two polaritonic peaks. The remainder of the peaks remain relatively unchanged compared to the reference spectrum obtained outside the cavity. As the light–matter coupling strength is increased, the splitting about the primary transition (i.e., the cavity frequency) grows, although it is asymmetric, with the LP peak shifting further away from the cavity frequency than the UP peak does. We also observe energetic shifts of the other peaks compared to the reference spectrum, and there are additional peaks that grow in intensity as the light–matter coupling strength is increased.

**Figure 9:**
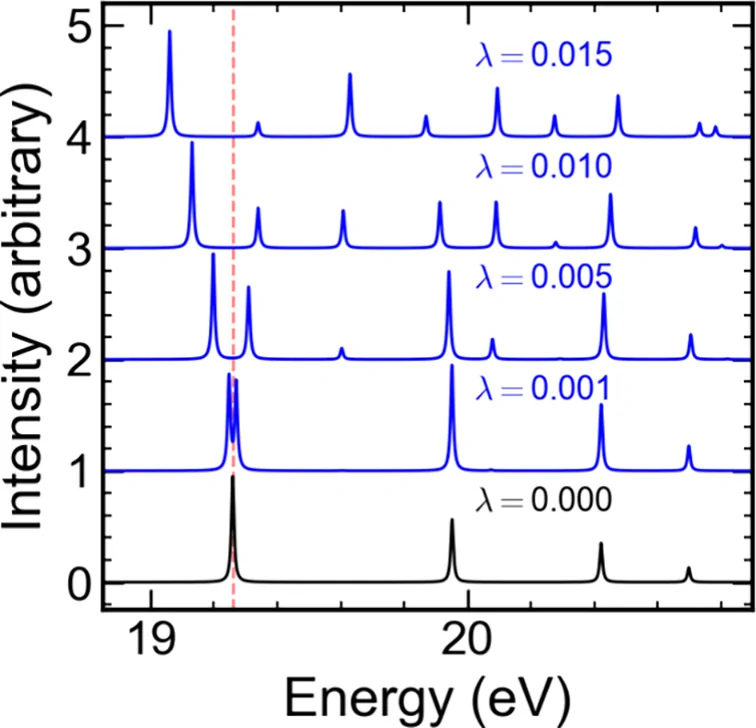
HeHHe^+^ vibronic progression both outside the cavity (black, labeled λ = 0.000) and inside the cavity with varying light–matter coupling strengths reported in atomic units. The vertical dashed red line indicates the cavity frequency ω. All transition energies and intensities are determined from a time-independent QED-NEO-CASCI calculation and are uniformly artificially broadened with a Lorentzian line shape that has a full width at half-maximum value of 0.01 eV. The spectra are scaled to have a maximum intensity of 0.95 and are offset vertically for clarity.

Both the shifts of the peaks present in the reference spectrum obtained outside the cavity and the growth of additional peaks can be explained by the coupling of the $\left|S_{0},\nu ,1\right\rangle$ manifold to the $\left|S_{1},\nu ,0\right\rangle$ manifold. For this analysis, we plot two of the polaritonic spectra (λ = 0.005 a.u. and λ = 0.010 a.u.), along with sticks indicating the energies of the relevant molecule plus photon states in Figure 10. For both light–matter coupling strengths, the splitting of the UP and LP peaks around the primary transition can be understood as the mixing of the $\left|S_{1},0,0\right\rangle$ and $\left|S_{0},0,1\right\rangle$ states. For the smaller light–matter coupling strength (λ = 0.005 a.u.), all peaks in the polaritonic spectrum correlate to one of the two manifolds of molecule plus photon states. The peaks that appear in the λ = 0.005 a.u. spectrum but have negligible intensity in the λ = 0.001 a.u. spectrum (primarily peaks at around 19.60 and 20.05 eV, but also minor peaks around 20.30 and 20.85 eV) correspond to transitions to the $\left|S_{0},\nu ,1\right\rangle$ manifold with an even number of quanta in the vibrational mode. Notably, outside the cavity, the $\left|S_{0},0\right\rangle \to \left|S_{0},\left\{2,4,6, ...\right\}\right\rangle$ transitions are dipole disallowed but Raman active. Inside the cavity, transitions to the $\left|S_{0},\nu ,1\right\rangle$ states with even ν gain oscillator strength because the bilinear coupling mixes this manifold with the $\left|S_{1},\nu ,0\right\rangle$ manifold via the molecular dipole operator. At the larger light–matter coupling strength (λ = 0.010 a.u.), the assignment of polaritonic peaks to molecule plus photon states is not as clear because the larger coupling mixes the two relevant manifolds of states more strongly. However, some general assignments can be made. For example, the two peaks around 19.9 and 20.1 eV likely result from mixing of $\left|S_{1},2,0\right\rangle$ and $\left|S_{0},4,1\right\rangle$ .

**Figure 10:**
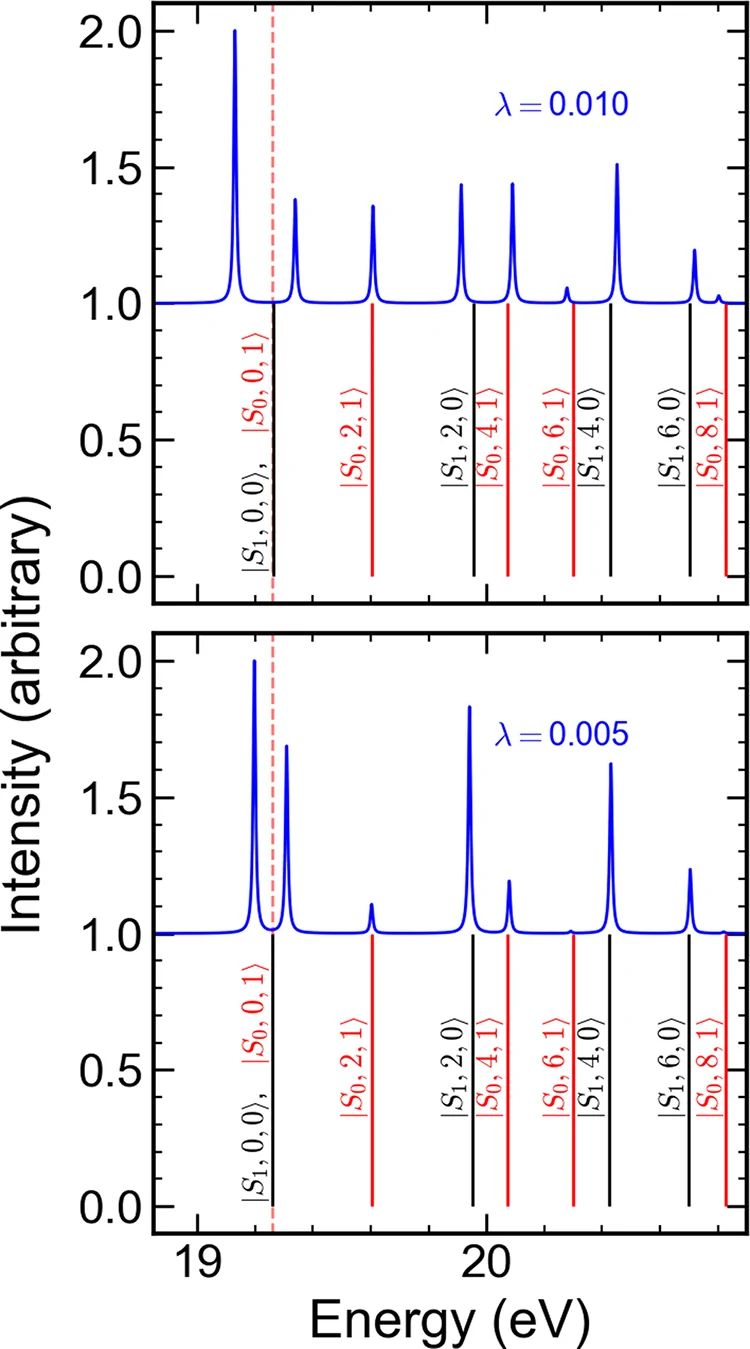
HeHHe^+^ vibronic progression inside the cavity with light–matter coupling strengths λ = 0.010 a.u. (top) and λ = 0.005 a.u. (bottom), in conjunction with an analysis of the peaks. The blue spectra are the same as the corresponding spectra in Figure 9. The vertical dashed red line indicates the cavity frequency ω. The black sticks show the energies of the states to which there are dipole allowed vibronic transitions from the $\left|S_{0},0,0\right\rangle$ ground state. The red sticks show the energies of the dipole disallowed vibrational transitions on the electronic ground state outside the cavity plus the energy of one cavity photon. $\left|S_{0},0,1\right\rangle$ is located at the same energy as $\left|S_{1},0,0\right\rangle$ .

Similar observations of transitions that are Raman active outside a cavity but present in the polaritonic absorption spectrum have been made by others in model systems, where it has been noted that the appearance of these peaks is a uniquely quantum mechanical phenomenon of polaritonic spectra.^78,79^ We did not observe these additional peaks in our previous NEO-TDCI simulations coupling a classical cavity mode to the vibronic excitations of HeHHe^+^.^54^ Our development of the QED-NEO-CASCI method, which is based on a fully quantum mechanical treatment of the total electron–proton–photon wave function, allows us to observe these features in polaritonic spectra from an ab initio quantum chemistry perspective. Similar to the previous work on model systems, we also find that observation of these peaks requires a fully quantum treatment, implying that they represent a nontrivial quantum phenomenon.

For this model system, the (4e,8o)-(1p,18o)-4 active spaces result in a full Hamiltonian dimension of 70 560 determinants. With our implementation, solving for 75 roots to capture this vibronic progression only requires ∼5.3 GB of RAM. It is also possible to calculate these spectra using QED-NEO-TDCI by applying a delta-pulse perturbation at *t* = 0 to the ground-state wave function.^80–82^ Calculating spectra in this manner avoids the computational bottleneck associated with solving for increasingly larger numbers of roots in time-independent calculations.^83^ Thus, QED-NEO-TDCI can be especially useful in applications involving dense manifolds of vibrational states between electronic states. For completeness, the same vibronic spectra for HeHHe^+^ calculated with QED-NEO-TDCI are provided in the . These spectra contain all the same peaks, but these peaks have wider line widths due to the signal processing associated with finite trajectories and a small shift toward higher energy resulting from the finite time step.

### 4.4 Water Dimer

Our final example is a water dimer, where the proton involved in the hydrogen bond experiences an anharmonic potential. We couple the cavity mode to the stretching mode of this quantized proton to illustrate how this coupling impacts the geometry of this molecule. Using the geometry, basis sets, and active spaces described in Section 3, this stretching mode has a frequency of ∼6200 cm^–1^, which is chosen as the cavity mode frequency. This frequency is much too high for a realistic O–H stretch because we are using a limited electronic active space and therefore lack the ability to capture sufficient electron–proton correlation to achieve accurate vibrational frequencies. However, this system still allows us to gain qualitative understanding of how coupling to a cavity can impact the geometries of hydrogen-bonded molecules. Additional calculations utilizing different electronic active spaces lead to qualitatively similar conclusions as those presented here. The results of these additional calculations are provided in and show that the stretching mode frequency decreases with a larger active space. It may be possible in the future to use the more sophisticated machinery of NEO-MRCI^42^ in QED contexts to provide more quantitative predictions.

The quantity of interest is the distance between the hydrogen bond donor oxygen and the quantized proton position expectation value, referred to as the O–H* distance, as shown in Figure 11. Outside the cavity, putting one quantum of energy into the anharmonic stretching mode lengthens this distance. This behavior is shown by the dashed lines in Figure 12, where the O–H* distance is around 0.01 Å larger in the first vibrationally excited state compared to the vibrational ground state. We will refer to the vibrational ground state and the first vibrationally excited state as $\left|\Psi_{0}\right\rangle$ and $\left|\Psi_{1}\right\rangle$ , respectively.

**Figure 11:**
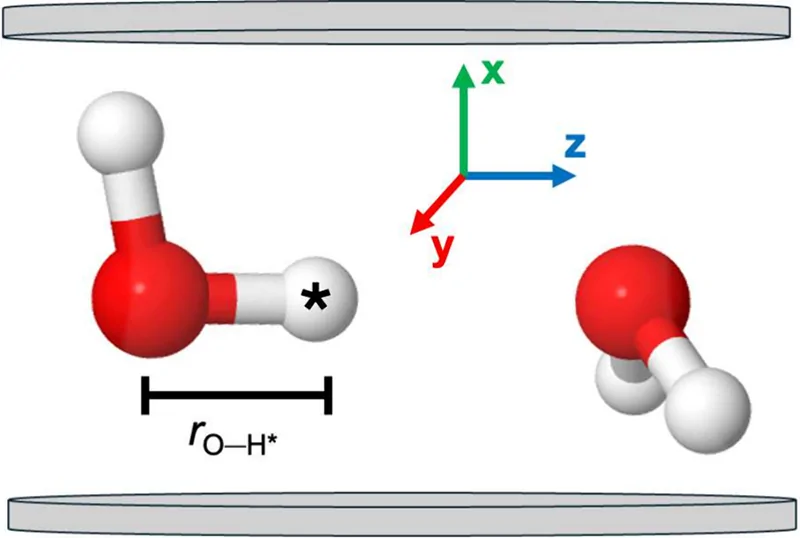
Schematic depiction of the water dimer coupled to a cavity mode. The hydrogen marked with an asterisk is treated quantum mechanically. The cavity mode is polarized in the *z*-direction.

**Figure 12:**
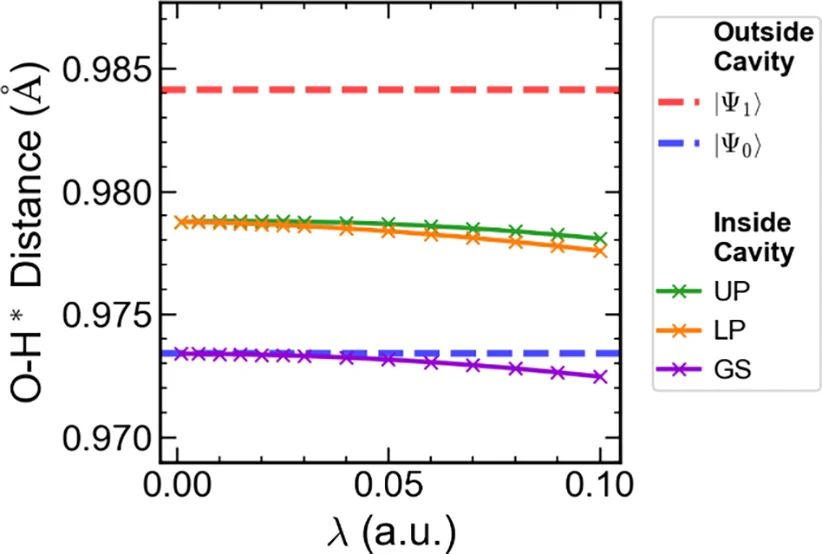
Distance between the donor oxygen and the quantum proton position expectation value in the water dimer. Outside the cavity, the vibrational ground state and the first excited vibrational state of the stretching mode are identified as $\left|\Psi_{0}\right\rangle$ and $\left|\Psi_{1}\right\rangle$ , respectively. Inside the cavity, the states are labeled as in Figure 3. The light–matter coupling strength λ is scanned from 0.001 to 0.10 in atomic units.

Next, we couple the cavity mode to this proton stretching mode. We scan the light–matter coupling strength from λ = 0.001 a.u. up to λ = 0.10 a.u. and plot the O–H* distance for the vibrational ground state as well as the UP and LP states. For the UP and LP states, at small values of the light–matter coupling strength, the O–H* distance is approximately halfway between the distances for the $\left|\Psi_{0}\right\rangle$ and $\left|\Psi_{1}\right\rangle$ states. This change in the average O–H* distance can be understood as a result of entanglement between exciting the vibrational mode versus exciting the cavity mode. The LP and UP states are both formed as roughly equally weighted linear combinations of $\left|\Psi_{0}^{\prime },1\right\rangle$ , which has the molecule in its vibrational ground state and one photon in the cavity mode, and $\left|\Psi_{1}^{\prime },0\right\rangle$ , which has the molecule in its vibrationally excited state and no photons in the cavity mode. One-dimensional slices of the proton density along the *z*-axis () confirm that the protonic densities associated with the polaritonic states can be interpreted as the roughly equal combination of the $\left|\Psi_{0}\right\rangle$ and $\left|\Psi_{1}\right\rangle$ protonic densities. Measuring the O–H* bond distance in these polaritonic states would therefore on average produce the $\left|\Psi_{0}\right\rangle$ distance and one photon in the cavity mode ∼50% of the time and the $\left|\Psi_{1}\right\rangle$ distance and no photons in the cavity mode ∼50% of the time, leading to the expectation value for this distance in both the LP and UP states approximately halfway between these values.

As the light–matter coupling strength increases, all three states shown experience a shortening of the O–H* distance. We note that this trend is in the same direction as was found previously at the QED-HF level of theory,^67^ although that study performed a full geometry optimization of all classical nuclei at each light–matter coupling strength, whereas our classical nuclei are fixed, and the only geometric changes arise from differences in the quantum protonic density. Nevertheless, it is reassuring that our quantum mechanical treatment of the hydrogen-bonding proton and a classical treatment of this proton at a lower level of theory produce the same geometric trends when the water dimer is coupled to a cavity mode. We also note that rotating the cavity polarization direction in either the *xz* or *yz* planes also leads to a shortening of the O–H* distance (see Section S5.5 of the Supporting Information). At larger light–matter coupling strengths, the O–H* distance for the UP and LP states is no longer the same. The one-dimensional slices of the protonic density along the *z*-axis (Figure S13) also show qualitative differences between the UP and LP states at larger light–matter coupling strengths. This analysis highlights the power of the QED-NEO-CASCI method for illustrating geometric changes under VSC.

## 5 Conclusions

In this work, we derived the working equations for QED-NEO-HF, QED-NEO-CASCI, and QED-NEO-TDCI and implemented them for molecular systems. All of these methods treat electrons, specified nuclei, and photons quantum mechanically. The interactions between these particles are treated at the mean-field level in QED-NEO-HF, whereas QED-NEO-CASCI and QED-NEO-TDCI incorporate correlation effects among all of the quantum degrees of freedom. QED-NEO-CASCI enables us to dissect the many-body correlated electron–proton–photon wave functions into contributions from easily interpretable molecule plus photon states. For the HeHHe^+^ system, we used QED-NEO-CASCI to study hydrogen tunneling under VSC. We also used QED-NEO-TDCI to simulate hydrogen tunneling dynamics in HeHHe^+^ and showed that coupling to a cavity mode dramatically changes the tunneling dynamics due to quantum mechanical energy exchange between the molecular vibrational and photonic modes. Additionally, we used QED-NEO-CASCI to investigate vibronic progressions under vibronic strong coupling, where the cavity mode was coupled to a vibronic excitation, and observed features in the polaritonic absorption spectrum that are present only in the Raman spectrum outside a cavity. For the water dimer system, we found that coupling the cavity mode to the vibrational stretch mode of the hydrogen-bonding proton alters the equilibrium geometry in both the ground and polaritonic states.

This work provides the foundation for many interesting future directions extending the QED-NEO methodology. Of particular interest is QED-NEO density functional theory, where, in addition to electron–proton^40,84–87^ and electron–photon^88–93^ correlation functionals, proton–photon correlation functionals will need to be developed. In terms of real-time dynamics of polaritonic systems, QED-NEO-TDCI alleviates limitations of RT-NEO-TDDFT coupled to a cavity mode^52,55^ by allowing the study of double excitations and multireference systems. The inclusion of multiple cavity modes and cavity-loss mechanisms characteristic of realistic plasmonic and nanocavity environments^17,53^ will be important for future calculations. The development of these capabilities will extend the applicability of NEO methods for investigating fundamental light–matter physics. Applications of these ab initio QED-NEO tools will provide insights into the coupled quantum mechanical behavior of electrons, nuclei, and photons critical to understanding polaritonic chemistry and, more generally, light–matter interactions.

## Supplementary Material





## Data Availability

The data and source code underlying this study are available in the published article, the Supporting Information, and on Zenodo at 10.5281/zenodo.21298676.

## Notes

Expressions for coherent state QED-NEO-CASCI, sample QED-NEO-HF calculations of HCN and HeHHe^+^, additional figures for QED-NEO-CASCI calculations of HeHHe^+^ under VSC, polaritonic spectra of HeHHe^+^ under vibronic strong coupling calculated with QED-NEO-TDCI, and additional details for the QED-NEO-CASCI water dimer calculations under VSC (PDF)
Movies showing time-dependent one-dimensional slices of the electronic and protonic density along with the time-dependent photon populations for the hydrogen tunneling trajectories in Section 4.2 (ZIP)
