# Supplementary material for: Cavity Quantum Electrodynamic Nuclear–Electronic Orbital Complete Active Space Configuration Interaction
Source: J Chem Theory Comput. 2026 Oct 24;22(21):11331–46. doi: 10.1021/acs.jctc.6c01384 (PMC13757757; doi:10.1021/acs.jctc.6c01384)
Supplement: Supplementary file 1 [file ct6c01384_si_001.pdf]

Supporting Information

Cavity Quantum Electrodynamic Nuclear–Electronic Orbital Complete Active Space Configuration Interaction

Scott M. Garner and Sharon Hammes-Schiffer*

Department of Chemistry, Princeton University, Princeton NJ 08544, United States

E-mail: shs566@princeton.edu

Contents

S1 Coherent State QED-NEO-CASCI Expressions **S3**

S2 QED-NEO-HF Sample Calculations **S4**

S2.1 HCN S4

S2.2 $HeHHe^+$: Squeezed Proton S6

S3 $HeHHe^+$ Coupling to Tunneling States **S8**

S3.1 $|\Psi'_n\rangle$ vs $|\Psi_n\rangle$ Comparison S8

S3.2 Time-Independent Eigenstates S9

S3.3 Independent Coupling Scheme S10

S3.4 Additional Time-Dependent Hydrogen Tunneling Trajectories S11

S3.5 Fourier Transforms of Time-Dependent Dipole Moments S13

S4 $HeHHe^+$ Vibronic Progression **S15**

S4.1 Time-Dependent Propagation S15

S5 Water Dimer Calculation Details **S17**

S5.1 Geometry S17

S5.2 Electronic Active Space S18

S5.3 Electronic Active Space Tests S19

S5.4 One-Dimensional Proton Density Slices S20

S5.5 Cavity Polarization Direction Scans S21

S1 Coherent State QED-NEO-CASCI Expressions

As stated in the main text, it is possible to formulate QED-NEO-CASCI using the coherent state representation of the photon mode. In this case, slightly different expressions are obtained for the modified core Hamiltonians, diagonal energy shift, and bilinear coupling. The modified core Hamiltonians are

$$h_{pq}^{\mathrm{e}'} = h_{pq}^{\mathrm{e}} - \frac{1}{2}q_{pq}^{\mathrm{e}} - \left\langle \hat{\boldsymbol{\mu}}_{\mathrm{Q}} \right\rangle d_{pq}^{\mathrm{e}} \tag{1a}$$

$$h_{PQ}^{\mathrm{p}'} = h_{PQ}^{\mathrm{p}} + \frac{1}{2}q_{PQ}^{\mathrm{p}} - \left\langle \hat{\boldsymbol{\mu}}_{\mathrm{Q}} \right\rangle d_{PQ}^{\mathrm{p}} \tag{1b}$$

where $\left\langle \hat{\boldsymbol{\mu}}_{\mathrm{Q}} \right\rangle$ is the quantum dipole operator expectation value. The diagonal energy contribution in the coherent state basis is

$$E_{\mathrm{diag}} = \frac{1}{2}\left\langle \hat{\boldsymbol{\mu}}_{\mathrm{Q}} \right\rangle^2 . \tag{2}$$

The bilinear coupling is given by

$$\hat{H}_{\mathrm{blc}} = -\sqrt{\frac{\omega}{2}}\left(\sum_{pq} d_{pq}^{\mathrm{e}}\hat{E}_{pq} + \sum_{PQ} d_{PQ}^{\mathrm{p}}\hat{E}_{PQ} - \left\langle \hat{\boldsymbol{\mu}}_{\mathrm{Q}} \right\rangle\right)\left(b^\dagger + b\right). \tag{3}$$

The remaining implementation details are identical to those for the photon number representation.

Although it has been shown for conventional QED-CASCI that the coherent state representation has many favorable properties compared to the photon number representation, we prefer to work in the photon number basis to avoid approximating $\left\langle \hat{\boldsymbol{\mu}}_{\mathrm{Q}} \right\rangle$. In conventional electronic CS-QED-CASCI, $\left\langle \hat{\boldsymbol{\mu}}_{\mathrm{Q}} \right\rangle \equiv \langle \hat{\boldsymbol{\mu}}_{\mathrm{e}} \rangle$. In previous conventional CS-QED-CASCI calculations, $\langle \hat{\boldsymbol{\mu}}_{\mathrm{e}} \rangle$ has been fixed to the expectation value of the dipole operator from a preceding CS-QED-HF calculation.[S1] For the resulting CS-QED-CASCI ground state, this value is

typically a good approximation, as the mean field and correlated ground state wavefunctions typically have similar dipole moments. However, for our $HeHHe^+$ calculations at the CS-QED-NEO-HF level of theory, the proton localizes near one of the helium atoms, leading to $\langle \hat{\boldsymbol{\mu}}_{\mathrm{Q}} \rangle \neq 0$. This localization is a well-known property of single-reference NEO methods.[S2] The QED-NEO-CASCI method, however, correctly produces a symmetric, bilobal ground state wavefunction with the proton delocalized between both helium atoms, and thus $\langle \hat{\boldsymbol{\mu}}_{\mathrm{Q}} \rangle = 0$. To avoid handling this mismatch, we chose to work in the photon number basis.

S2 QED-NEO-HF Sample Calculations

S2.1 HCN

We performed calculations on HCN at the geometry given in Table S1. We utilized the cc-pVTZ[S3] electronic and PB5-F[S4] protonic basis sets. The cavity mode frequency was $\omega = 0.1$ a.u. and was coupled to the molecule with light–matter coupling strength $\lambda = 0.1$ a.u. polarized along the z axis, which is the HCN axis. We performed HF, QED-HF, NEO-HF, and QED-NEO-HF calculations, where the QED calculations used both the CS and PN photon representations. The absolute energies from these calculations are plotted in Figure S1. Although the absolute energies are not meaningful, the relative absolute energies computed with the different methods are physically meaningful.

Table S1: The HCN geometry used for the calculations shown in Figure S1. The proton was treated quantum mechanically.

Atom	x	y	z
N	0.000000	0.000000	-1.00000
C	0.000000	0.000000	0.15824
H	0.000000	0.000000	1.22844

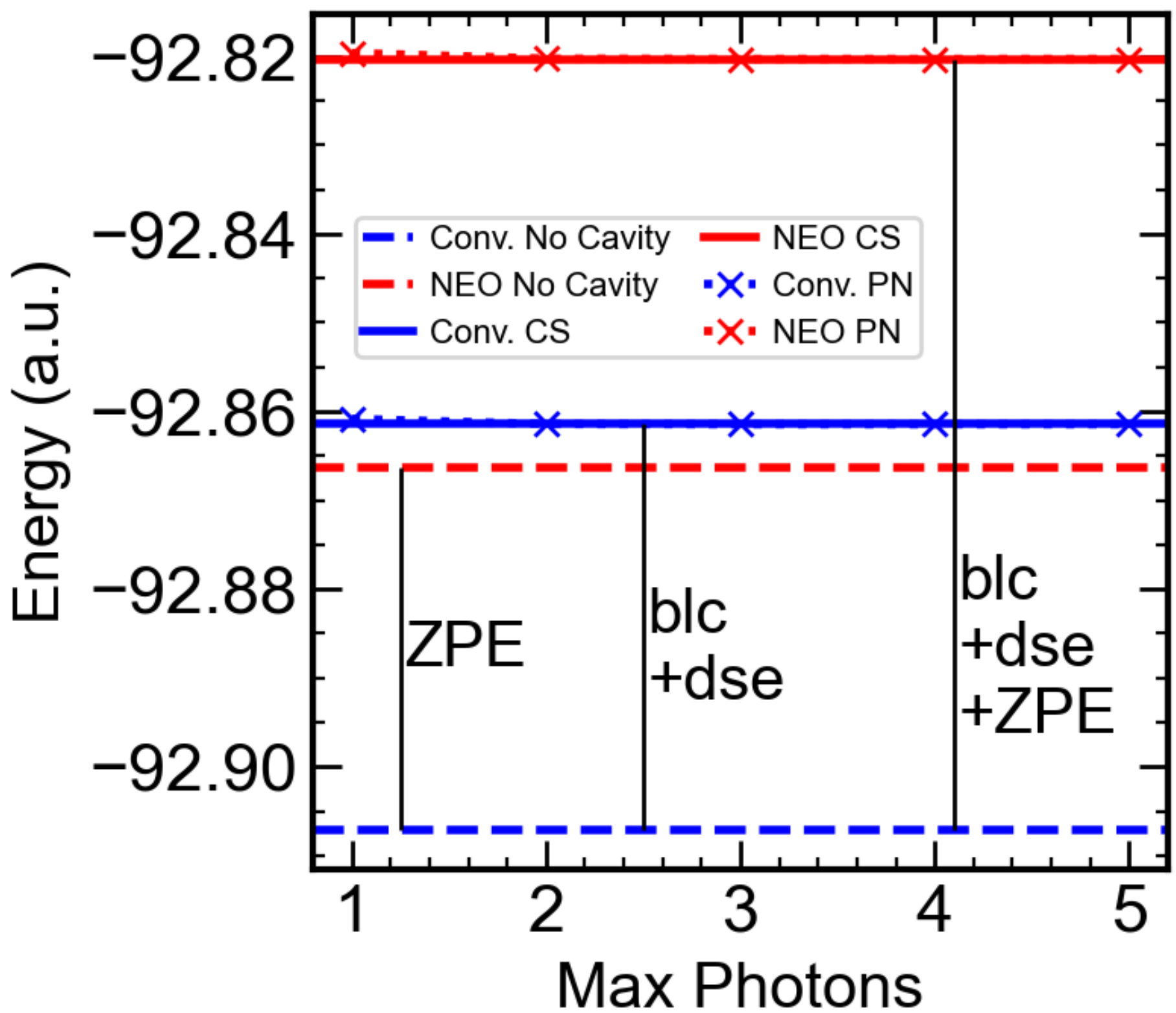

Figure S1: HF, NEO-HF, QED-HF, and QED-NEO-HF absolute energies for HCN. Blue lines indicate conventional electronic calculations, where all nuclei are treated as point charges, while red lines are NEO calculations with the proton treated quantum mechanically. Dashed lines represent calculations performed outside the cavity, solid lines represent calculations performed inside the cavity with the CS photon representation, and dotted lines with X symbols represent calculations performed inside the cavity with the PN photon representation. The convergence of the PN photon representation calculations as a function of the maximum number of photons considered is shown. The energy difference between the blue dashed and red dashed lines is the zero-point energy (ZPE) from quantizing the proton. The energy difference between the blue dashed and blue solid lines is the additional energy from the bilinear coupling (blc) and dipole self-energy (dse). The energy difference between the blue dashed line and the solid red line arises from all three contributions (blc, dse, and ZPE). Both PN-QED-HF and PN-QED-NEO-HF quickly converge to their respective CS-QED-HF and CS-QED-NEO-HF energies with respect to the maximum number of photons considered.

S2.2 HeHHe$^+$: Squeezed Proton

To show how coupling to a cavity can direcly influence nuclear delocalization at the QED-NEO-HF level of theory, we performed calculations on HeHHe$^+$. We used a He–He distance of 1.8 Å, where the potential experienced by the proton within the conventional Born–Oppenheimer approximation is a single well, in contrast to the double-well potential at the He–He distance of 2.2 Å used in the main text. We used the cc-pVTZ[S3] electronic and the PB5-F[S4] protonic basis sets. One protonic and associated electronic basis set was placed at the origin, with the He atoms placed symmetrically 0.9 Å from the origin along the z axis. The cavity frequency was $\omega = 0.1$ a.u. and was polarized along the z axis.

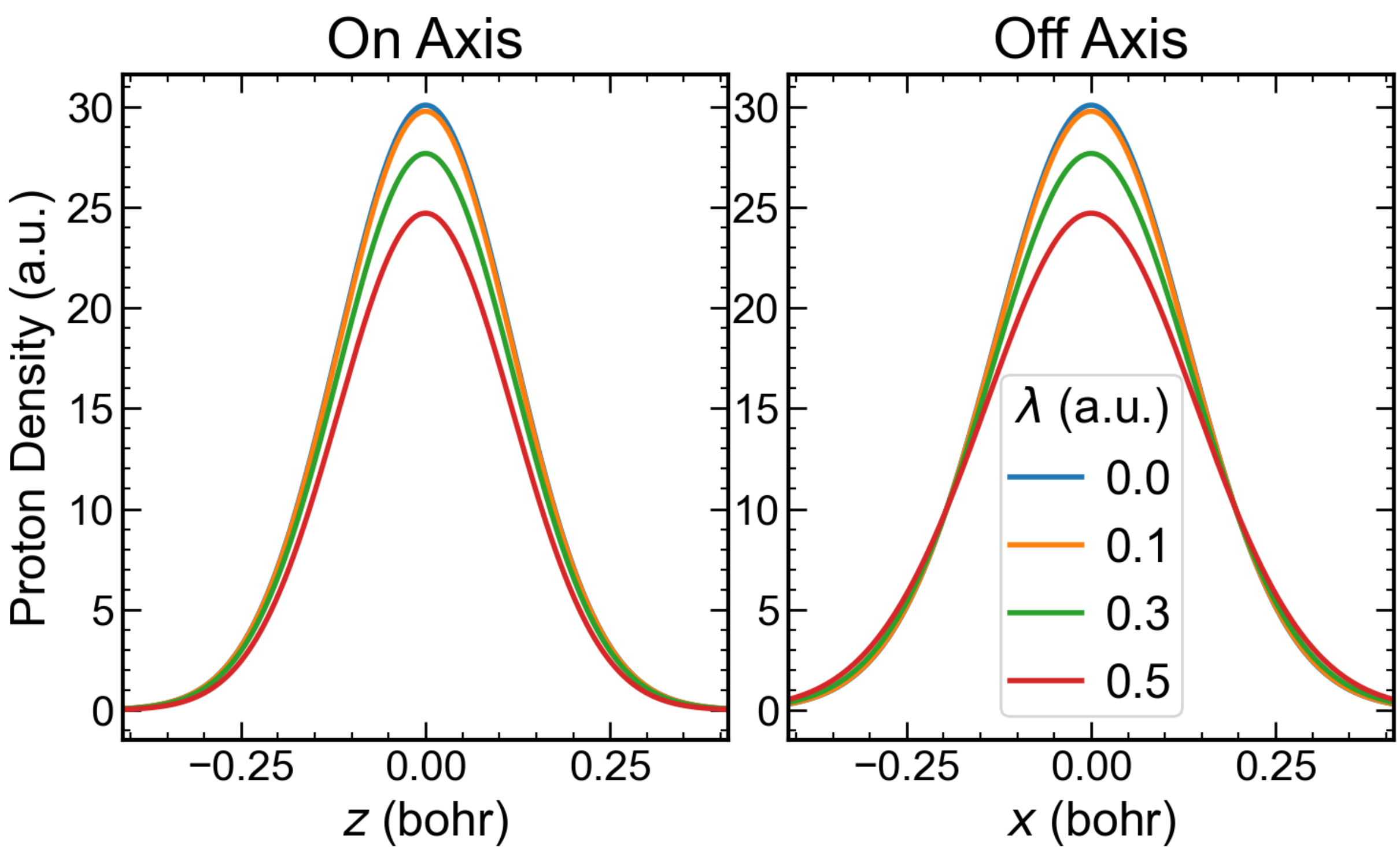

Figure S2: One-dimensional slices of the proton density in HeHHe$^+$ obtained from a CS-QED-NEO-HF calculation outside the cavity ($\lambda = 0.0$ a.u.) and inside the cavity with three different light–matter coupling strengths.

One-dimensional slices of the proton density along the z and x axes obtained from a CS-QED-NEO-HF calculation are shown in Figure S2. Because the molecule has no dipole moment, CS-QED-NEO-HF and PN-QED-NEO-HF calculations produce identical results

for this system. As the light–matter coupling strength is increased, the proton delocalizes in the xy plane, as indicated by the slight increase in the width of the proton density along the x axis, and the proton becomes more localized along the z axis, as indicated by the slight decrease in the width of the proton density along this axis. This squeezing of the proton has been observed previously.[S5] Here, the squeezing can be understood as a result of the self-dipole term. The proton is minimizing its dipole variance in the direction of the light–matter coupling, as the variance along this axis contributes to an energy penalty arising from the $\frac{1}{2}(\boldsymbol{\lambda} \cdot \hat{\boldsymbol{\mu}})^2$ self-dipole term.

S3 $HeHHe^+$ Coupling to Tunneling States

S3.1 $|\Psi'_n\rangle$ vs $|\Psi_n\rangle$ Comparison

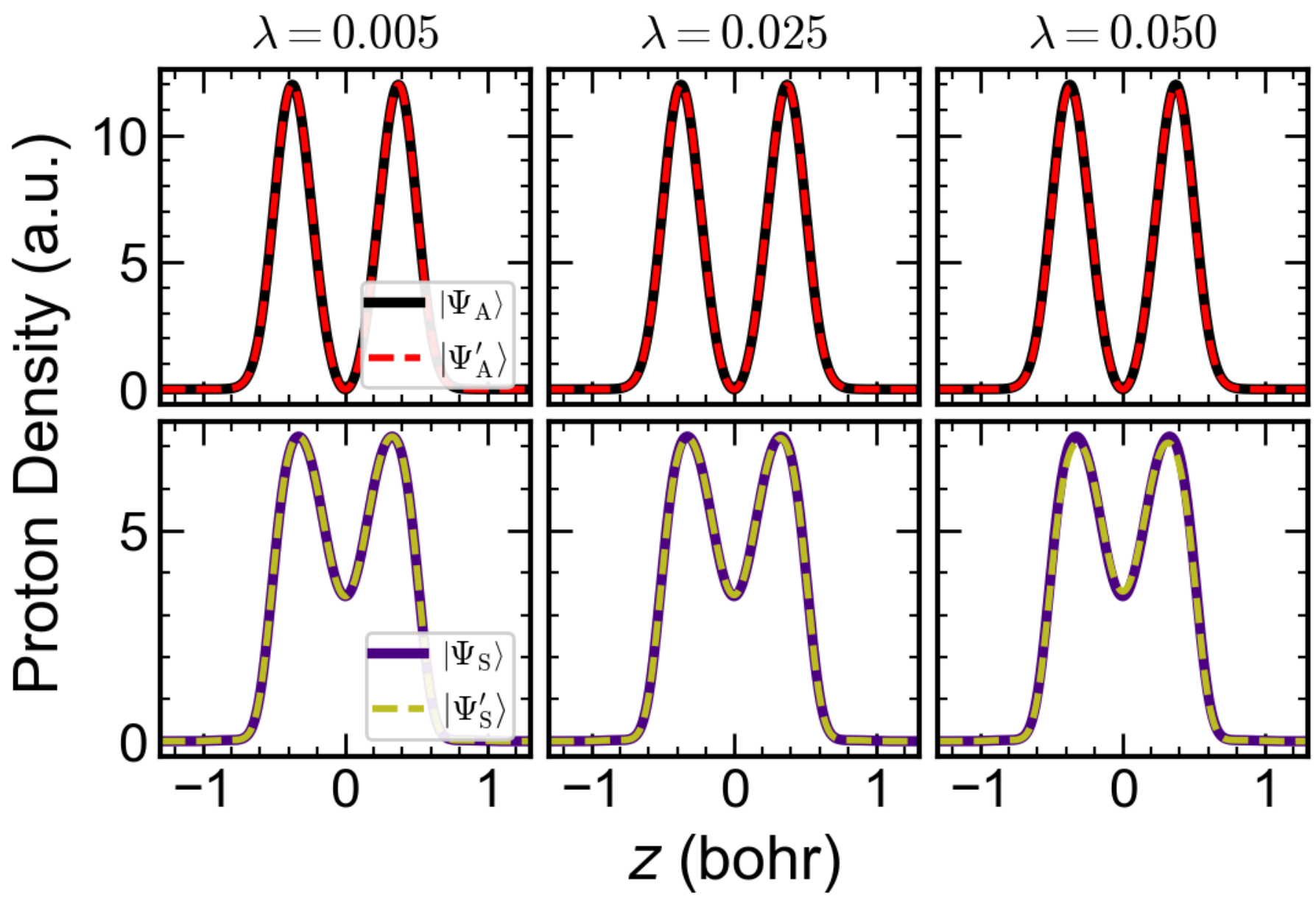

Figure S3: One-dimensional slices of the proton density in $HeHHe^+$ showing the qualitative similarities between $|\Psi'_n\rangle$ and $|\Psi_n\rangle$ (see the main text for definitions). The $|\Psi'_n\rangle$ densities are from a QED-NEO-CASCI calculation with $N_{\mathrm{max}}^{\mathrm{ph}} = 0$, while the $|\Psi_n\rangle$ densities are from an analogous NEO-CASCI calculation. The light–matter coupling strengths labeling each column are given in atomic units.

S3.2 Time-Independent Eigenstates

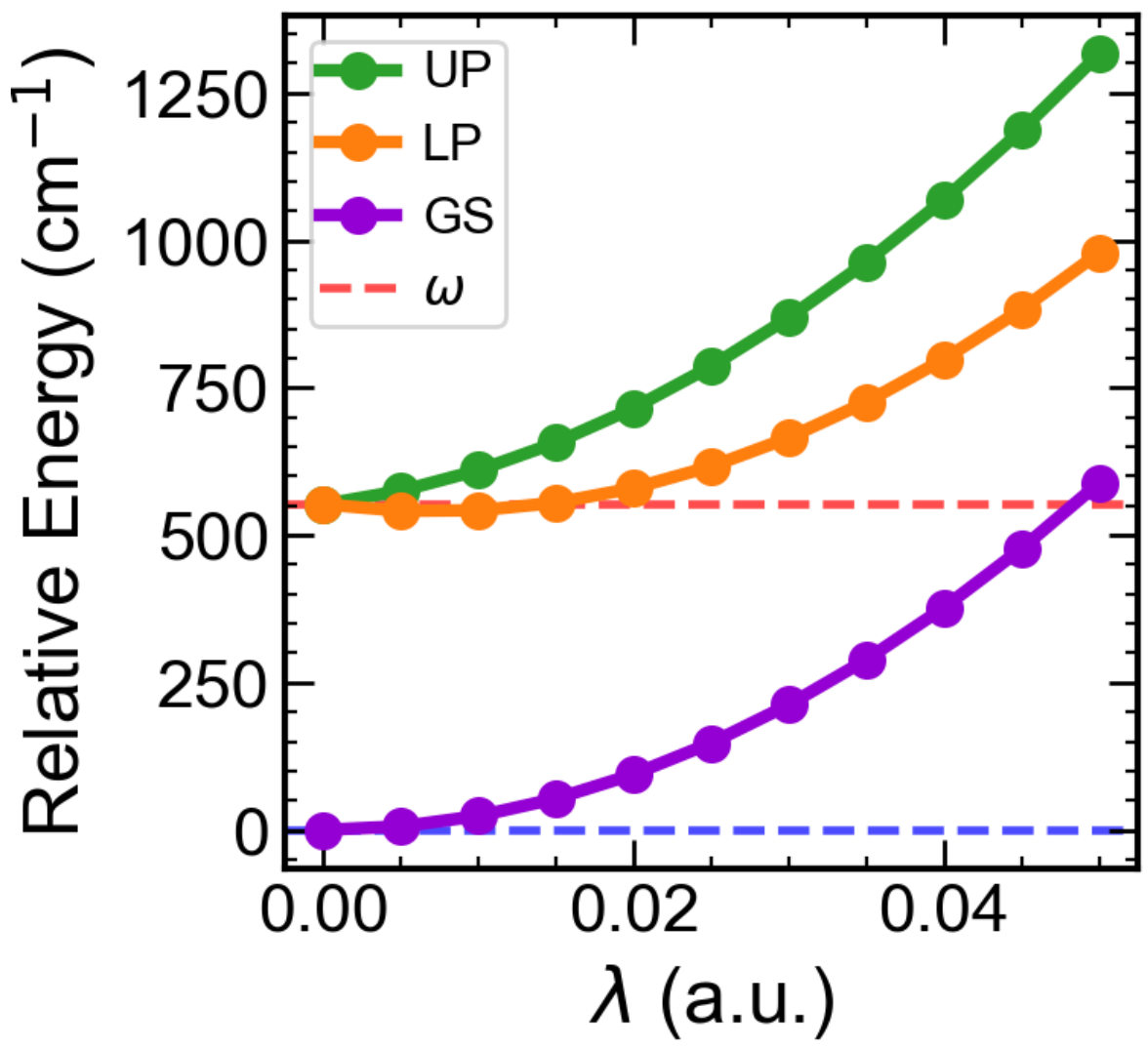

Figure S4: Energies of the ground (purple), lower polaritonic (orange), and upper polaritonic (green) states for $HeHHe^+$ as a function of the light–matter coupling strength λ when the cavity frequency (red dashed line) is tuned to the tunneling splitting (see main text Section 4.1). The main text Figure 4 results from subtracting the purple curve from the orange and green curves. The absolute energy of the cavity-free ground state, indicated by the dashed blue line, is -5.8002224599 Hartree. Because these states have no permanent dipole moment, the approximate quadratic upward curvature is primarily a result of the dipole self-energy term, which is quadratic in λ.

S3.3 Independent Coupling Scheme

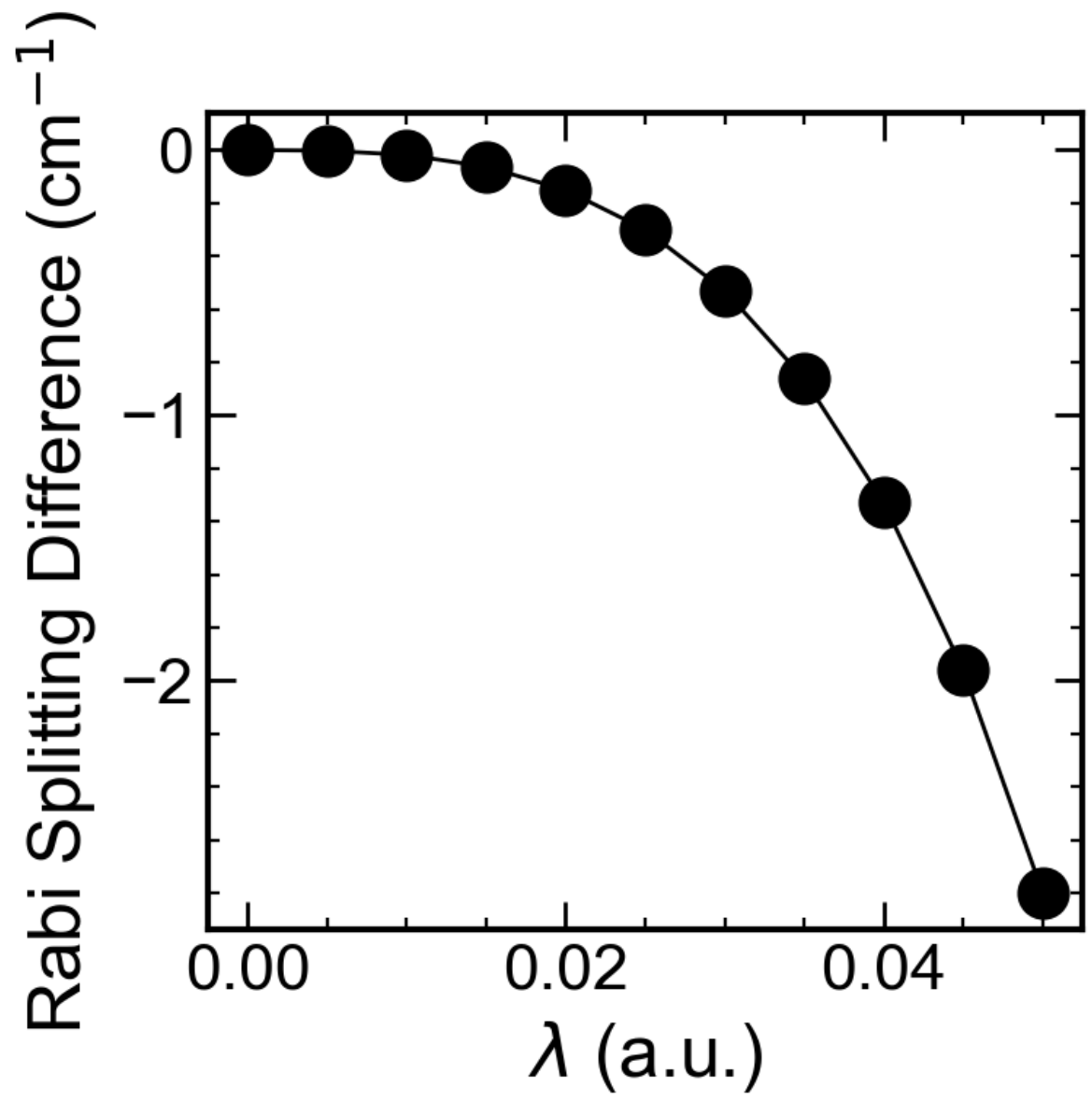

Figure S5: Difference in Rabi splittings when the cavity mode is coupled to both the electrons and the proton versus the sum of the Rabi splittings when the cavity mode is coupled only to the electrons or the proton. The plot shows $\Omega_{\mathrm{ep}} - (\Omega_{\mathrm{e}} + \Omega_{\mathrm{p}})$ (see main text for definitions).

S3.4 Additional Time-Dependent Hydrogen Tunneling Trajectories

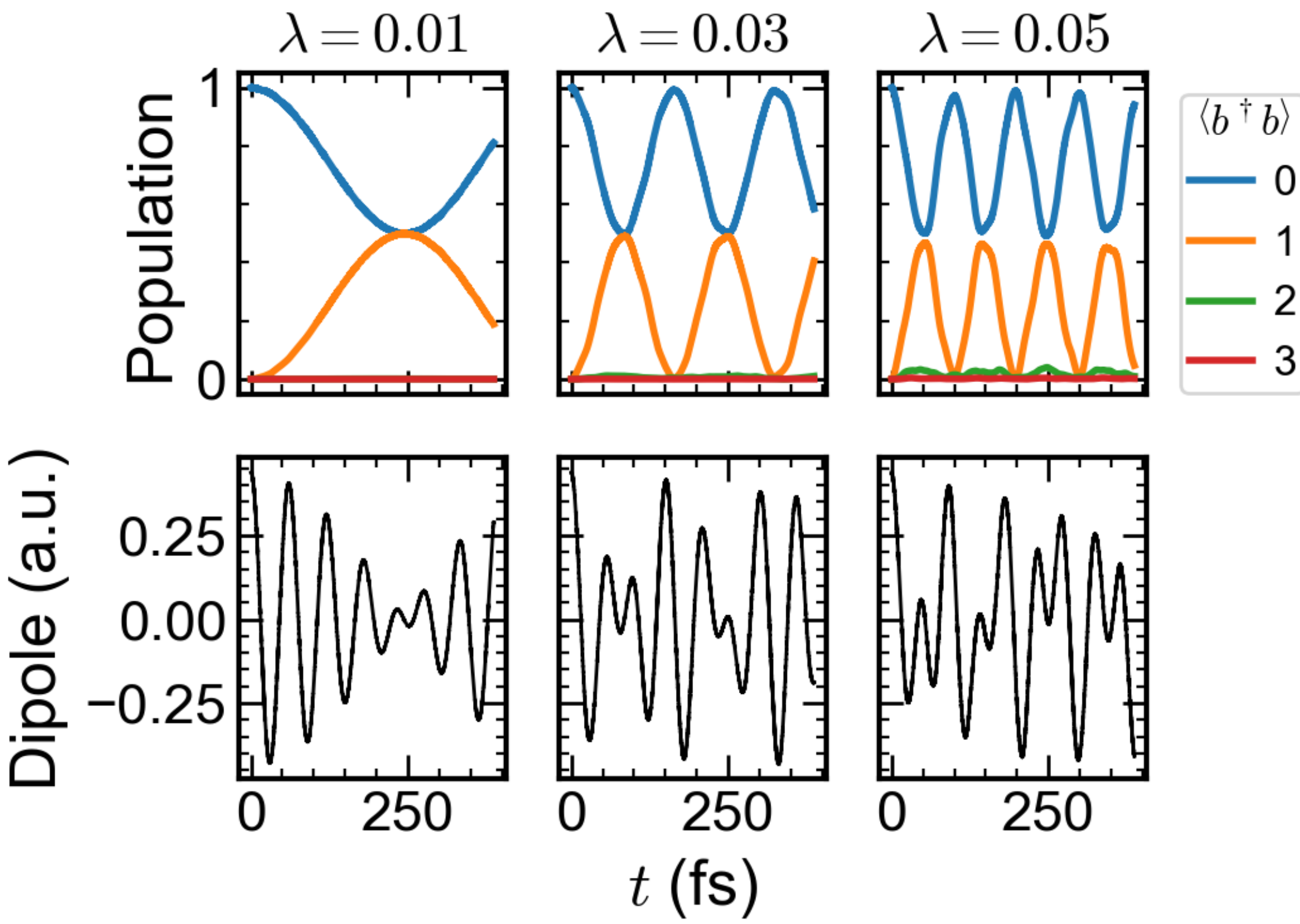

Figure S6: Time-dependent photon number state populations (top row) and molecular dipole moments (bottom row) for QED-NEO-TDCI trajectories of $HeHHe^+$ initialized from the localized state $|\Psi(t=0)\rangle = \frac{1}{\sqrt{2}}(|\Psi_S, 0\rangle + |\Psi_A, 0\rangle)$. Although the cavity can be populated by up to 4 photons, only the populations up to 3 photons are shown for clarity, as the 4 photon number state gains negligible population in all trajectories. The light–matter coupling strength λ is given in atomic units.

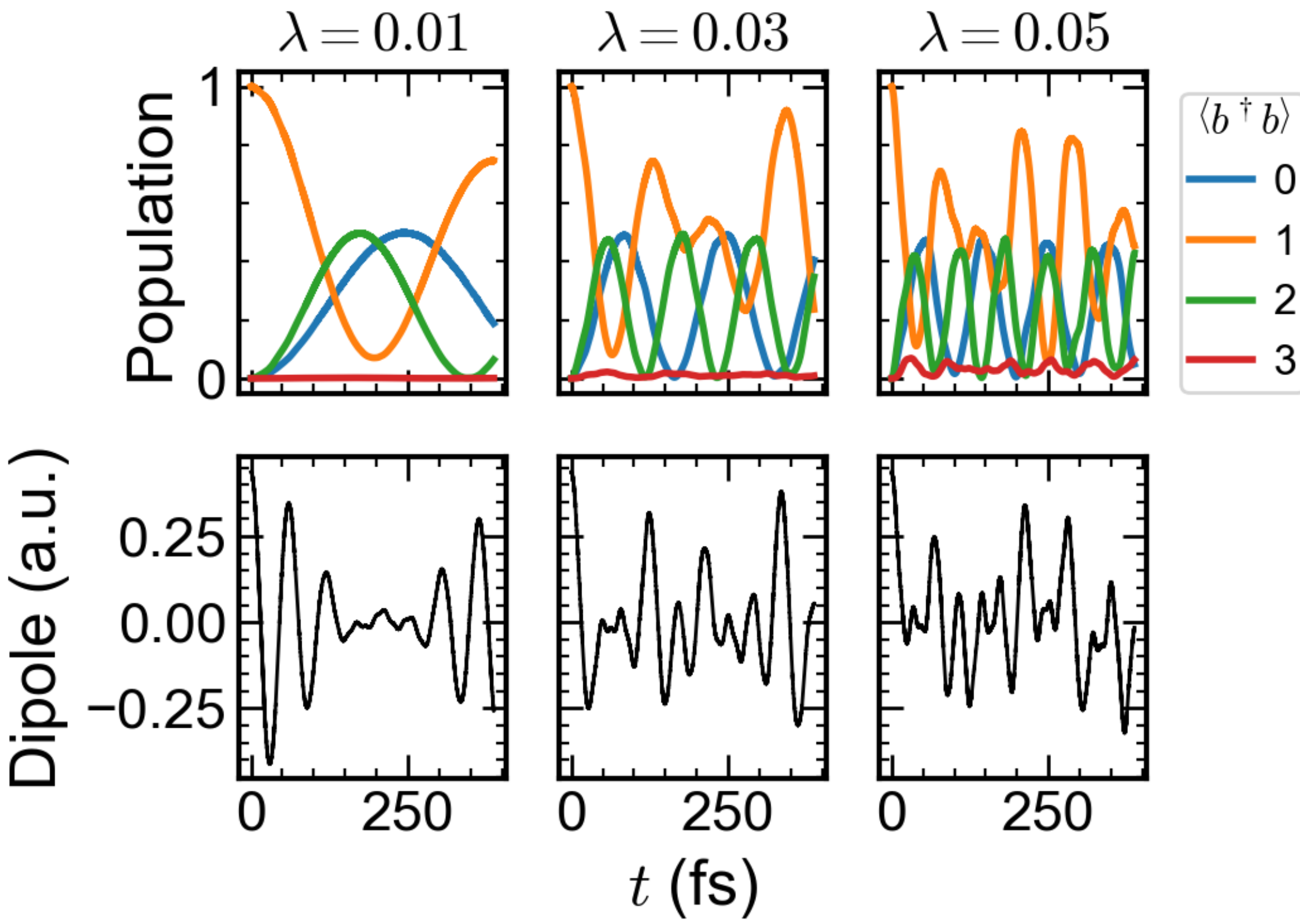

Figure S7: Time-dependent photon number state populations (top row) and molecular dipole moments (bottom row) for QED-NEO-TDCI trajectories of $HeHHe^+$ initialized from the localized state $|\Psi(t=0)\rangle = \frac{1}{\sqrt{2}}(|\Psi_S, 1\rangle + |\Psi_A, 1\rangle)$. Although the cavity can be populated by up to 4 photons, only the populations up to 3 photons are shown for clarity, as the 4 photon number state gains negligible population in all trajectories. The light–matter coupling strength λ is given in atomic units.

S3.5 Fourier Transforms of Time-Dependent Dipole Moments

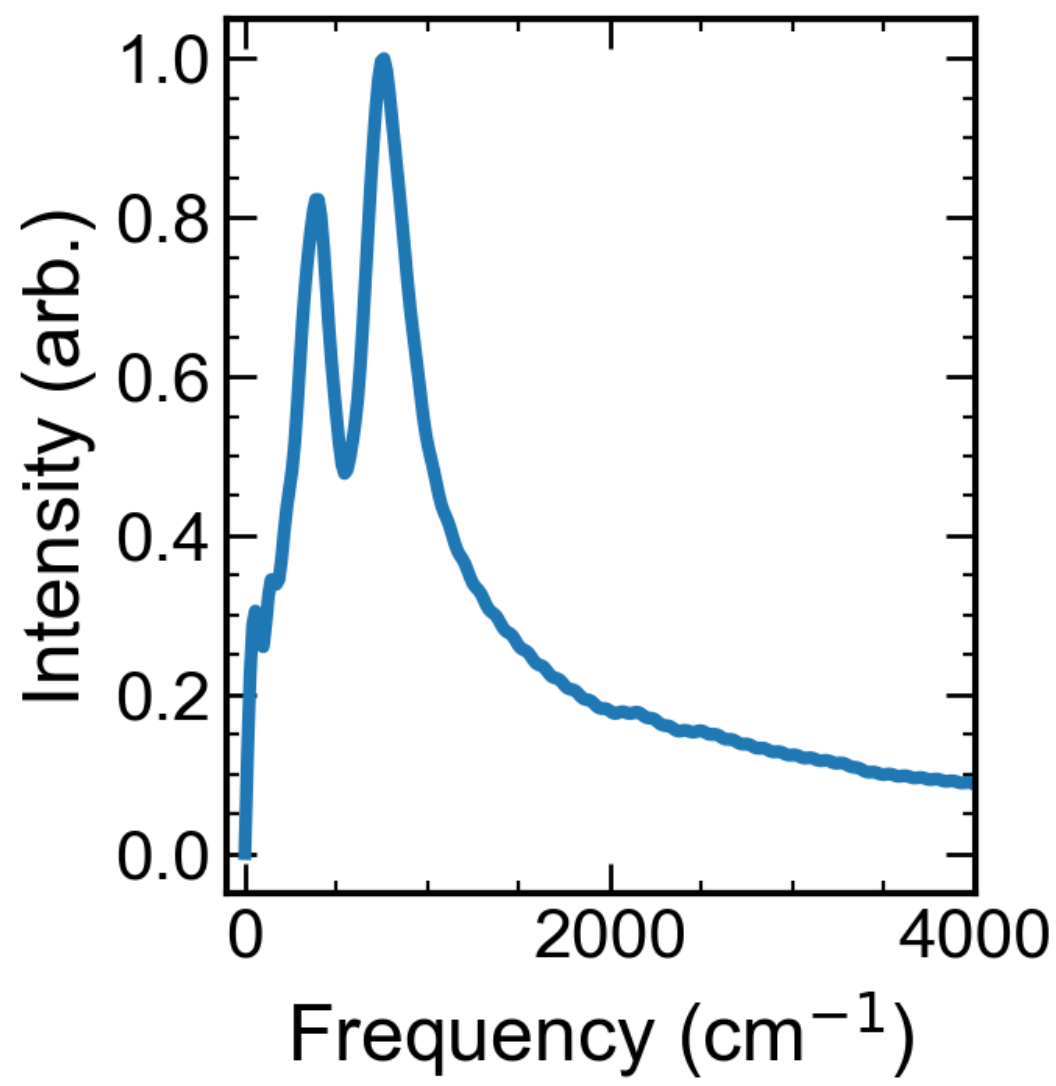

Figure S8: Absolute value of the Fourier transform of the time-dependent dipole moment from the tunneling trajectory initialized with 0 cavity photons (main text Figure 7, lower left). The time-dependent dipole was dampened with an exponential factor of $e^{-\Gamma t}$ with $\Gamma = 5 \times 10^{-4}$. The broadness of the peaks and ringing artifacts in the signal are a result of the finite trajectory.

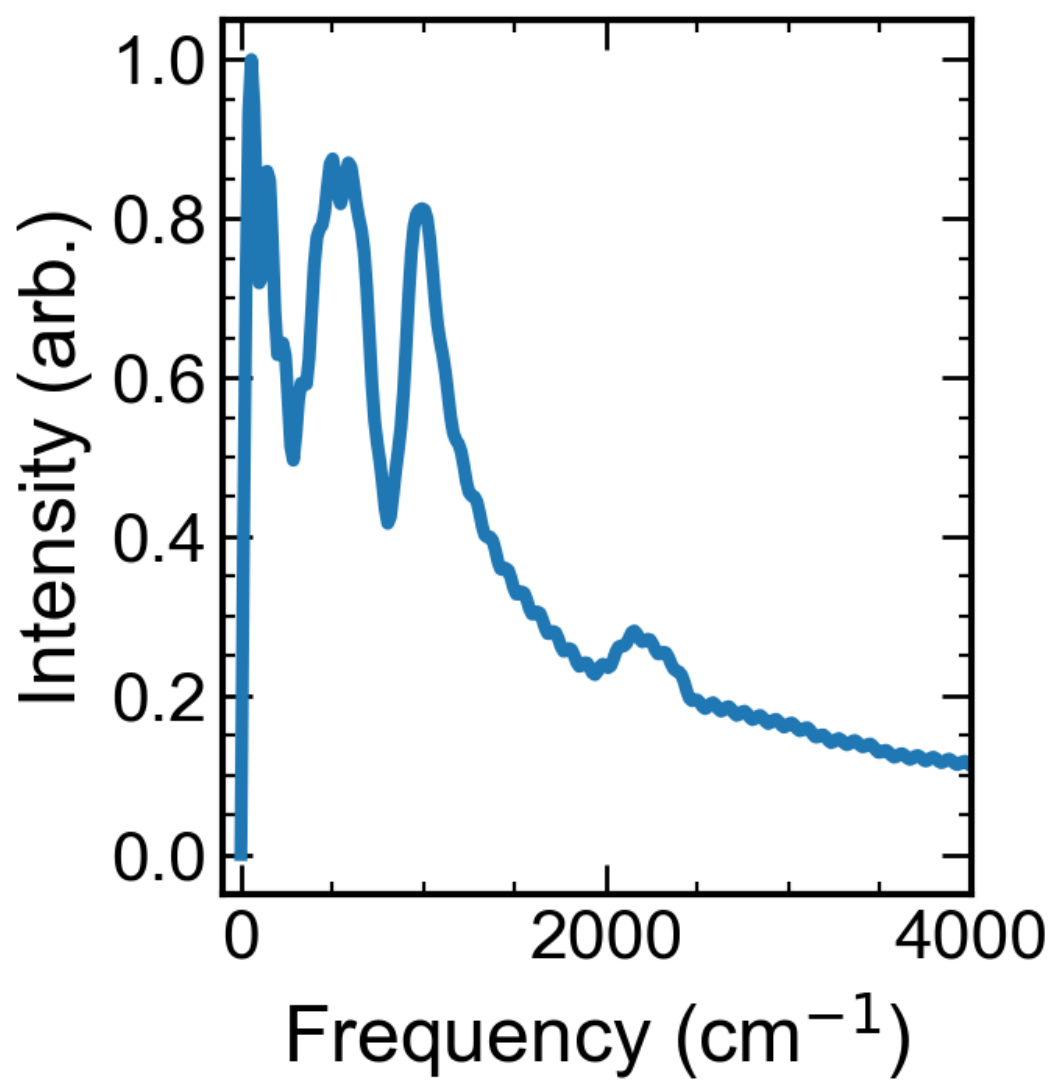

Figure S9: Absolute value of the Fourier transform of the time-dependent dipole moment from the tunneling trajectory initialized with 0 cavity photons (main text Figure 7, lower right). The time-dependent dipole was dampened with an exponential factor of $e^{-\Gamma t}$ with $\Gamma = 5 \times 10^{-4}$. The broadness of the peaks and ringing artifacts in the signal are a result of the finite trajectory.

S4 $HeHHe^+$ Vibronic Progression

S4.1 Time-Dependent Propagation

Here, we present the computational details and results for the vibronic coupling QED-NEO-TDCI trajectories, which produce equivalent results as the time-independent QED-NEO-CASCI calculations discussed in Section 4.3 of the main text. For all trajectories, a time step of 0.01 a.u. was used, and the wavefunctions were propagated for a total of 8,000 a.u. ($\approx$ 194 fs). A delta pulse electric field was applied to the molecular degrees of freedom for only the first time step with an intensity of 0.001 a.u. along the z axis. To calculate the spectra, we took the imaginary component of the Fourier transform of the time-dependent molecular dipole moment.[S6,S7] The time-dependent molecular dipole moment was dampened with an exponential dampening factor $e^{-\Gamma t}$ with $\Gamma = 6 \times 10^{-4}$.

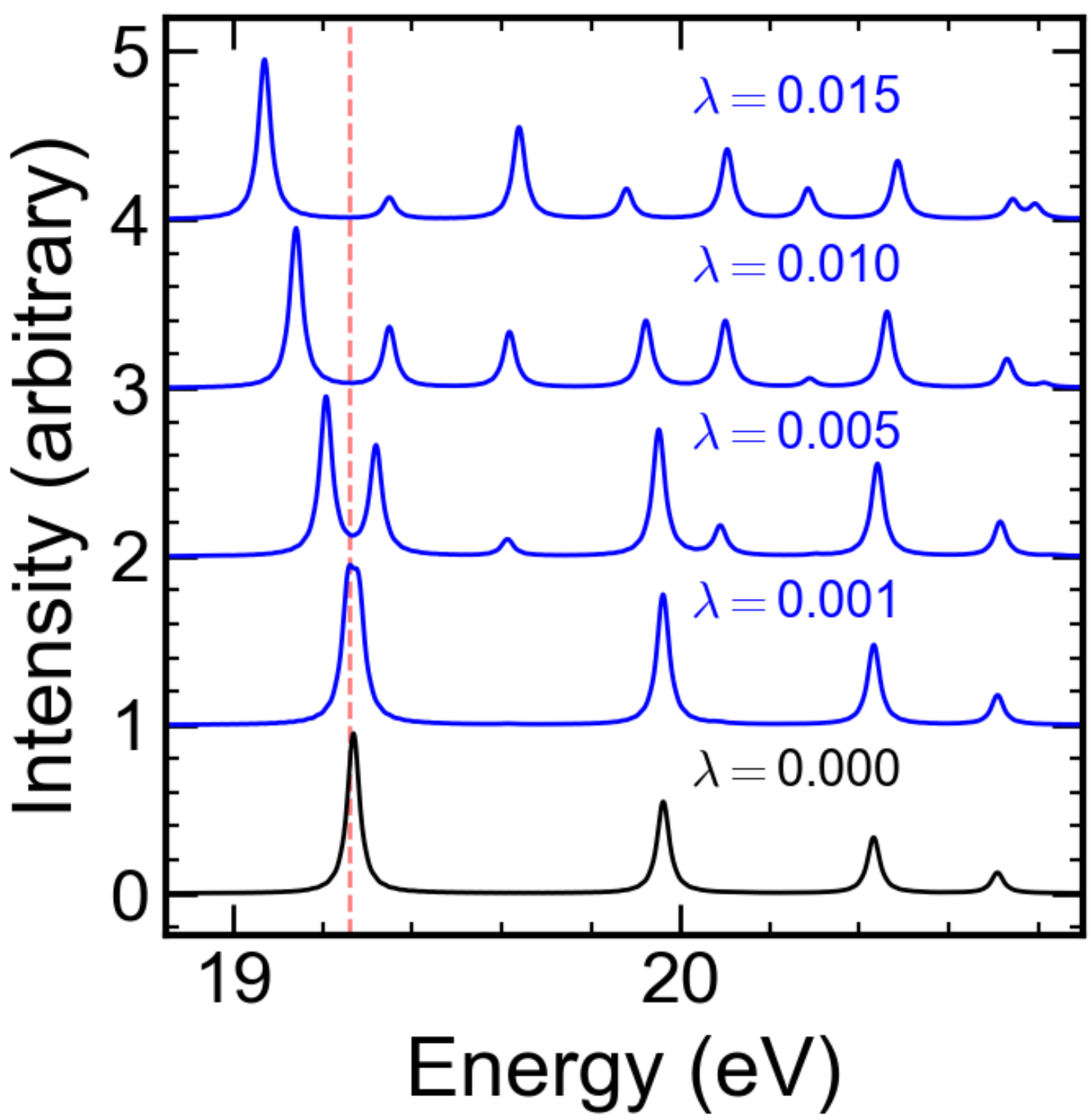

Figure S10: Analagous vibronic coupling spectra to Figure 9 of the main text but calculated with QED-NEO-TDCI instead of time-independent QED-NEO-CASCI. $HeHHe^+$ vibronic progression both outside the cavity (black, labeled $\lambda = 0.000$) and inside the cavity with varying light–matter coupling strengths reported in atomic units. The vertical dashed red line indicates the cavity frequency ω. The spectra are scaled to have maximum intensity of 0.95 and are offset vertically for clarity.

S5 Water Dimer Calculation Details

S5.1 Geometry

Table S2: Water dimer geometry used in the main text. The bold H was treated quantum mechanically.

Atom	x	y	z
O	0.0000000000	0.0000000000	-1.5196665107
**H**	0.0000000000	0.0000000000	-0.5492441137
H	-0.9448404590	0.0000000000	-1.7130773835
O	0.0000000000	0.0000000000	1.3905673542
H	0.5495726410	-0.7537463565	1.6437687373
H	0.5495726410	0.7537463565	1.6437687373

S5.2 Electronic Active Space

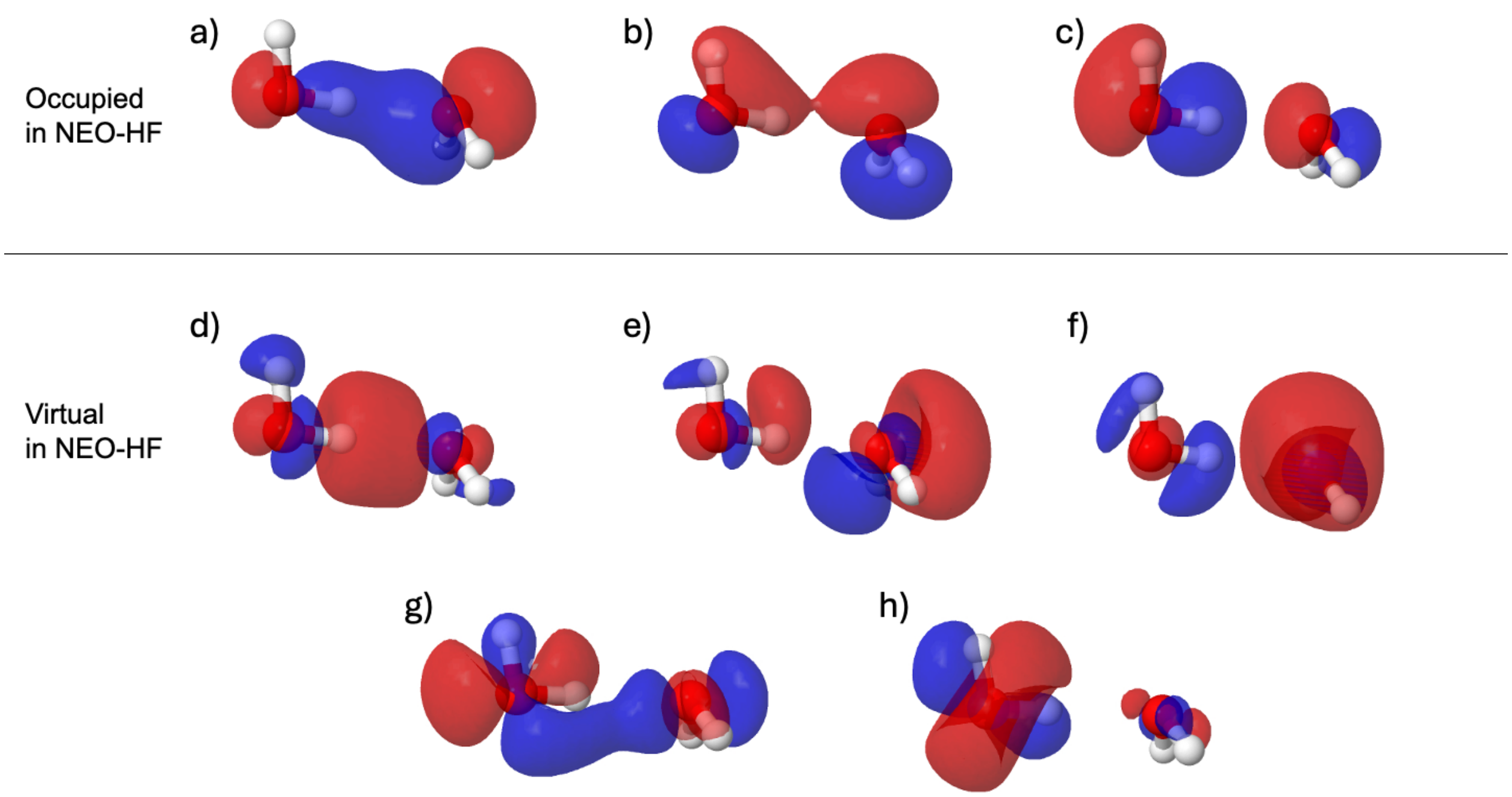

Figure S11: The orbitals that comprise the electronic active spaces in the QED-NEO-CASCI calculations for the water dimer in the main text and Figure S12. The orbitals in the top row are occupied at the NEO-HF level of theory, while the orbitals in the bottom row are unoccupied. The active space used in the main text is composed of orbitals shown in (a)–(f).

S5.3 Electronic Active Space Tests

In addition to the (6e,6o)-(1p,46o)-6 calculations for the water dimer in the main text, we performed calculations utilizing both a smaller, (4e,6o)-(1p,46o)-6, and larger, (6e,8o)-(1p,46o)-6, electronic active space. All other details of the calculations remain identical to the (6e,6o)-(1p,46o)-6 calculations in the main text. The (6e,8o)-(1p,46o)-6 calculation produces a vibrational excitation energy of $\sim$5900 cm^{-1}, which is slightly closer to a realistic O-H stretching frequency than the $\sim$6200 cm^{-1} obtained from the (6e,6o)-(1p,46o)-6 calculation.

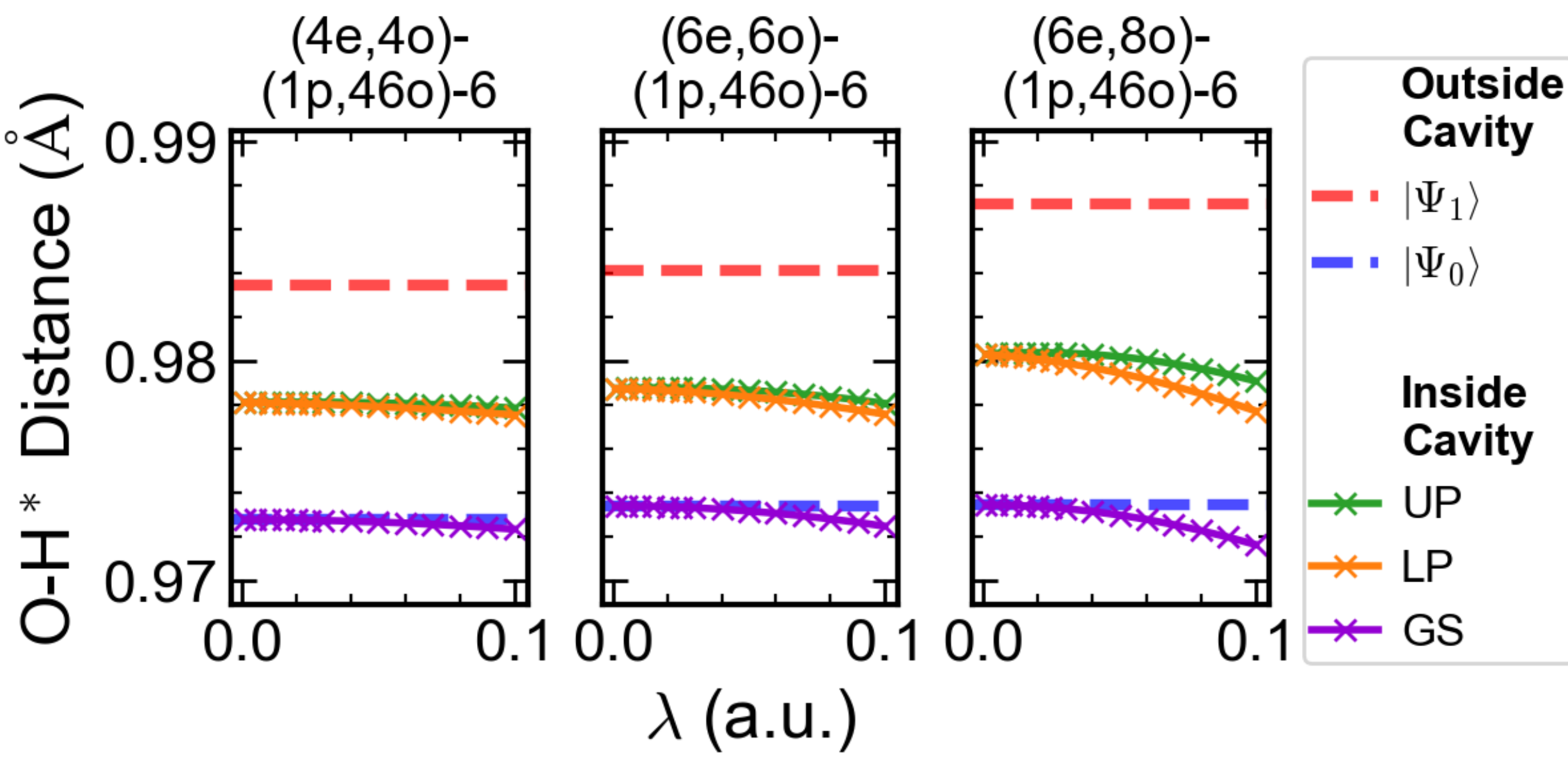

Figure S12: Distance between the donor oxygen and the quantum proton position expectation value in the water dimer for three different electronic active spaces. The center panel is equivalent to the main text Figure 12. Outside the cavity, the vibrational ground state and the first excited vibrational state of the stretching mode are identified as $|\Psi_0\rangle$ and $|\Psi_1\rangle$, respectively. Inside the cavity, the states are labeled as in Figure 3. The light–matter coupling strength λ is scanned from 0.001 to 0.10 in atomic units. The (4e,4o)/(1p,46o) active space is composed of the orbitals shown in (b)–(e) of Figure S11. The (6e,6o)/(1p,46o) active space is composed of the orbitals shown in (a)–(f) of Figure S11. The (6e,8o)/(1p,46o) active space is composed of all orbitals shown in Figure S11.

S5.4 One-Dimensional Proton Density Slices

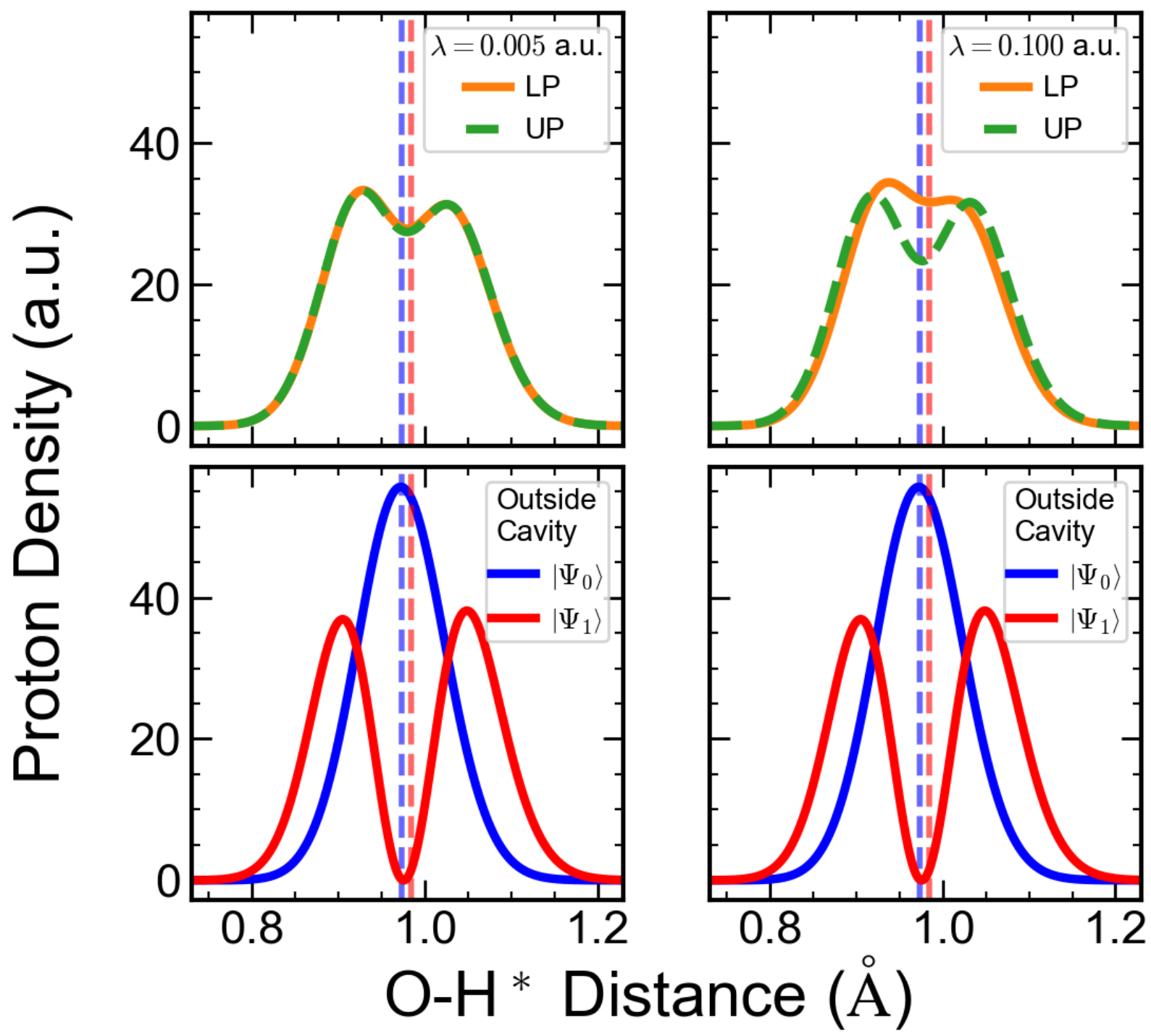

Figure S13: One-dimensional slices of the proton density along the z axis for the water dimer calculations discussed in the main text. The lower panels are identical and show the proton density slices for the system outside the cavity, with vertical dashed lines showing the proton position expectation value for the respective states. Although the conventional geometry optimization constrained the oxygen nuclei and proton involved in the hydrogen bond to be collinear along the z axis, in the NEO-HF calculation at that geometry, the quantum proton position expectation value deviates slightly off this axis. As a result, the $|\Psi_1\rangle$ proton density slice does not strictly go to zero in the region of the node. The upper panels show one-dimensional slices of the proton density for the lower and upper polaritonic states when coupled to the cavity mode with frequency $\omega = 0.028296785$ Hartree and light–matter coupling strength indicated in the legends.

S5.5 Cavity Polarization Direction Scans

To test the orientation dependence of the calculations in the main text, we have performed calculations rotating the cavity polarization direction in the xz and yz planes (as defined in Table S2). All other details of the calculations (e.g., geometry, basis set, and active space) remain identical to those of the calculations with the cavity polarized along the z axis in the main text. We rotate the polarization in 15° increments up to 90° in the xz plane. For rotations in the yz plane, we stop at 75° because at 90° the cavity polarization direction and O–H* stretching mode are strictly orthogonal. Analogous plots to Figure 12 of the main text for each of these orientations are shown in Figure S14 for the rotation in the xz plane and Figure S15 for the rotation in the yz plane. At all cavity polarization directions tested, we observe a shortening of the ground state O–H* distance in the cavity, although the quantitative magnitude is reduced as the polarization rotates away from the z axis. When the cavity polarization is rotated far from the z axis (e.g., $> 60^{\circ}$), the relative weights of $|\Psi_0', 1\rangle$ and $|\Psi_1', 0\rangle$ in the two polariton states are no longer roughly equal, leading to diverging O–H* expectation values for these states.

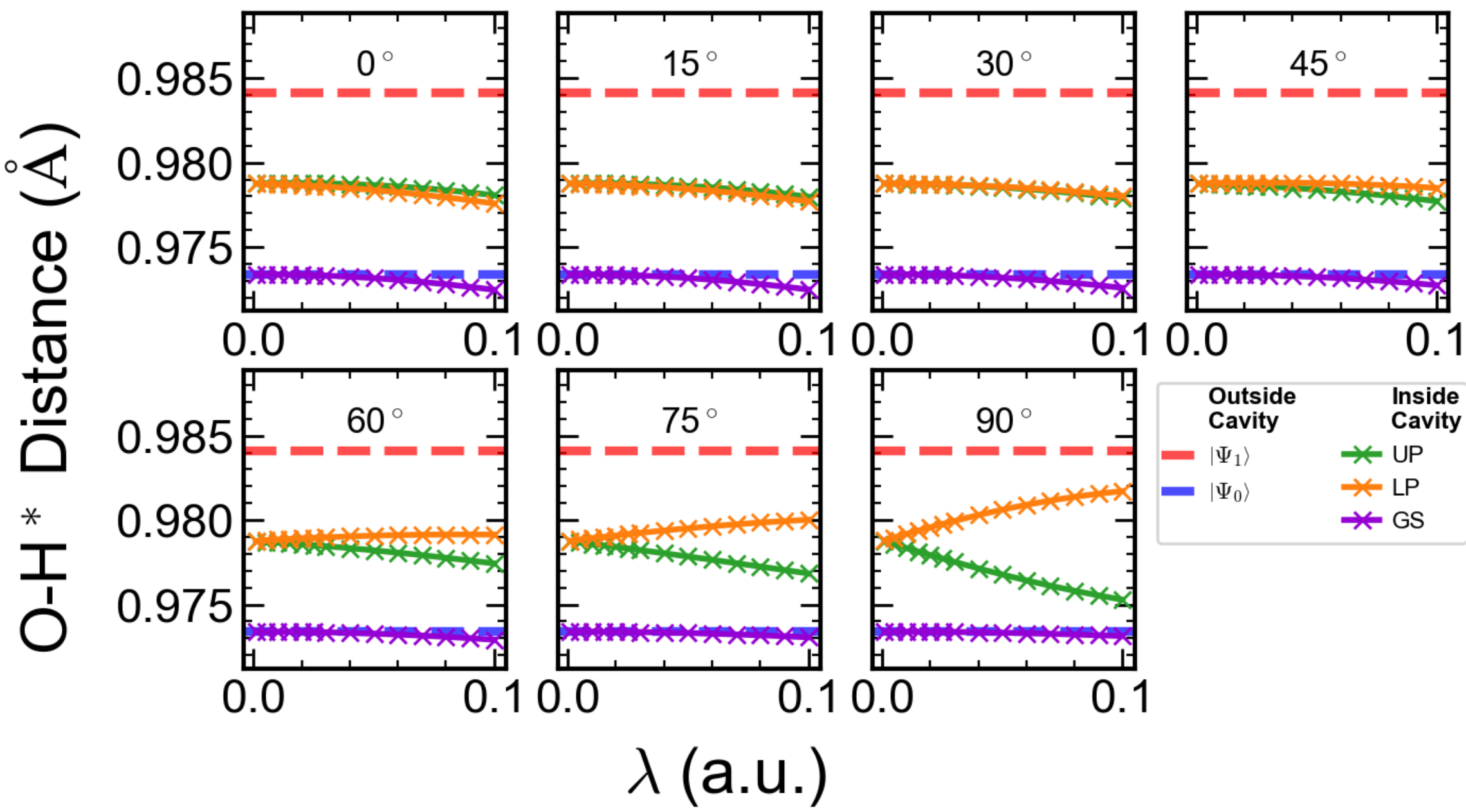

Figure S14: Distance between the donor oxygen and the quantum proton position expectation value in the water dimer when the cavity polarization is rotated in the xz plane. 0° is aligned with the z axis (equivalent to Figure 12 of the main text), while 90° is aligned along the x axis. Outside the cavity, the vibrational ground state and the first excited vibrational state of the stretching mode are identified as $|\Psi_0\rangle$ and $|\Psi_1\rangle$, respectively. Inside the cavity, the states are labeled as in Figure 3. The light–matter coupling strength λ is scanned from 0.001 to 0.10 in atomic units.

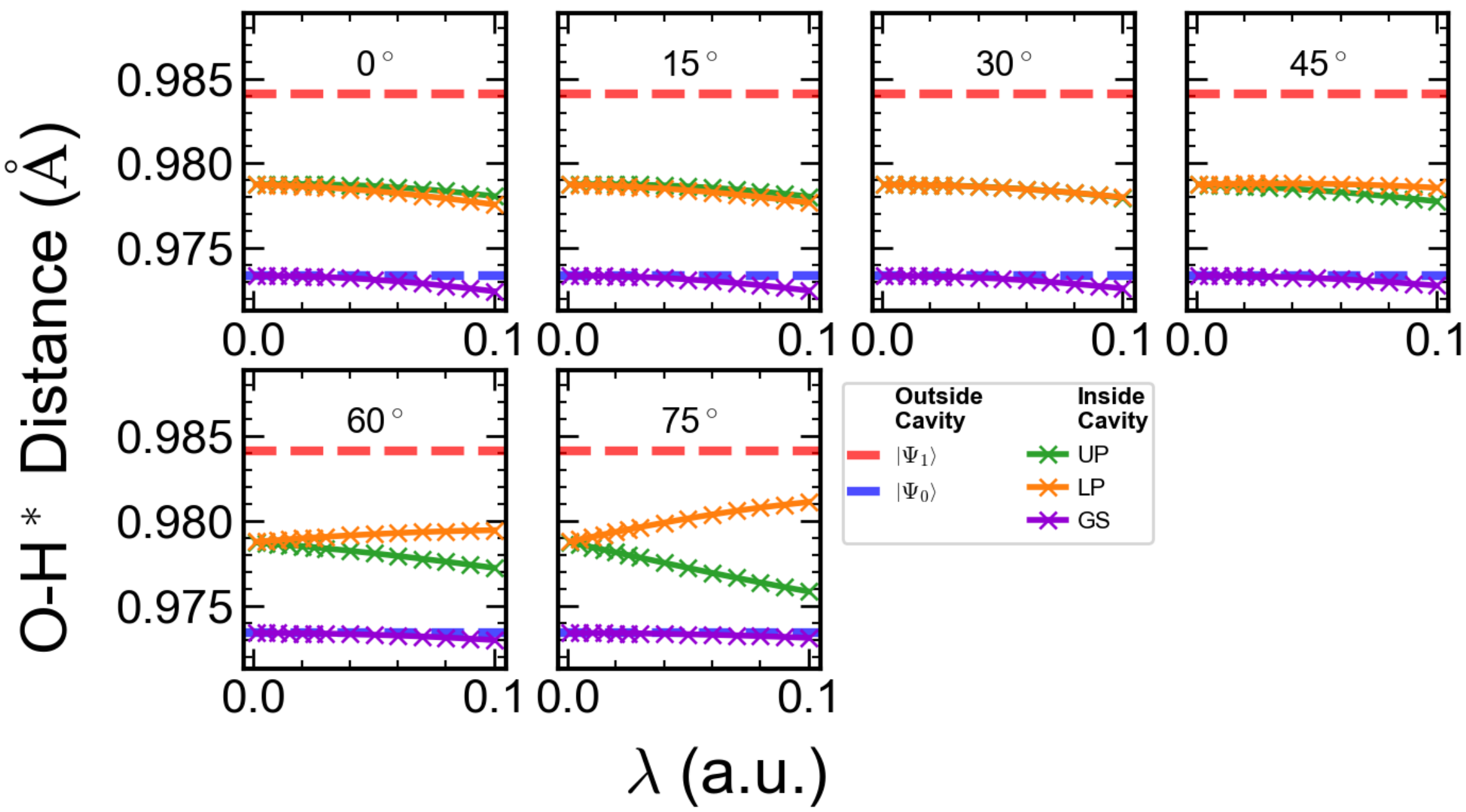

Figure S15: Distance between the donor oxygen and the quantum proton position expectation value in the water dimer when the cavity polarization is rotated in the yz plane. 0° is aligned with the z axis (equivalent to Figure 12 of the main text). Outside the cavity, the vibrational ground state and the first excited vibrational state of the stretching mode are identified as $|\Psi_0\rangle$ and $|\Psi_1\rangle$, respectively. Inside the cavity, the states are labeled as in Figure 3. The light–matter coupling strength λ is scanned from 0.001 to 0.10 in atomic units.

References

[S1] Vu, N.; Mejia-Rodriguez, D.; Bauman, N. P.; Panyala, A.; Mutlu, E.; Govind, N.; Foley, J. J. Cavity quantum electrodynamics complete active space configuration interaction theory. *J. Chem. Theory Comput.* **2024**, *20*, 1214–1227.

[S2] Yu, Q.; Hammes-Schiffer, S. Nuclear-electronic orbital multistate density functional theory. *J. Phys. Chem. Lett.* **2020**, *11*, 10106–10113.

[S3] Hehre, W. J.; Ditchfield, R.; Pople, J. A. Self—consistent molecular orbital methods. XII. Further extensions of Gaussian—type basis sets for use in molecular orbital studies of organic molecules. *J. Chem. Phys.* **1972**, *56*, 2257–2261.

[S4] Yu, Q.; Pavošević, F.; Hammes-Schiffer, S. Development of nuclear basis sets for multicomponent quantum chemistry methods. *J. Chem. Phys.* **2020**, *152*, 244123.

[S5] Li, T. E.; Paenurk, E.; Hammes-Schiffer, S. Squeezed protons and infrared plasmonic resonance energy transfer. *J. Phys. Chem. Lett.* **2024**, *15*, 751–757.

[S6] Goings, J. J.; Lestrange, P. J.; Li, X. Real-time time-dependent electronic structure theory. *WIREs Comput. Mol. Sci.* **2018**, *8*, e1341.

[S7] Li, X.; Govind, N.; Isborn, C.; DePrince III, A. E.; Lopata, K. Real-time time-dependent electronic structure theory. *Chem. Rev.* **2020**, *120*, 9951–9993.
